# Mending the Achilles heels of titin in cardiac and musculoskeletal disease

**DOI:** 10.1007/s12551-026-01411-4

**Published:** 2026-02-14

**Authors:** Ines Martinez-Martin, Roberto Silva-Rojas, Jorge Alegre-Cebollada

**Affiliations:** 1 Centro Nacional de Investigaciones Cardiovasculares (CNIC), Madrid, Spain; 2 Department of Physics, King’s College London, London, UK

**Keywords:** Muscle, Heart, Sarcomere, Mechanics, Cardiomyopathy, Myopathy

## Abstract

Titin, the largest known human protein, spans the sarcomere from Z-disk to M-line and is central to muscle elasticity, force transmission, and structural integrity. Maybe not surprisingly, accumulated evidence over the last years shows that titin, despite its titanic size, is not devoid of molecular *Achilles heels* that can lead to dysfunction and disease. In this review, we summarize the fundamental roles of titin in muscle mechanics, mechanosignaling, and physiology as well as in genetic and acquired disorders of cardiac and skeletal muscle. We discuss the current understanding of how mutations and posttranslational processing (dys)regulate titin, while highlighting gaps of knowledge regarding underlying molecular mechanisms. Finally, we analyze emerging experimental titin-cleavage models that are uncovering novel pathways of titin-based pathogenesis, positioning the protein not only as a central player in myocyte biomechanics but also as a determinant of pathological tissue remodeling. A main driving force in the field is to exploit the accumulated knowledge on titin to find new avenues for therapeutic intervention in cardiac and musculoskeletal disease.

## Introduction

Muscle function is fundamental to life — driving movement, maintaining posture, and powering the rhythmic beating of the heart. The proper function of muscle depends not only on the muscle’s ability to actively generate force at sarcomeres (Gautel and Djinović-Carugo 2016) but also on its finely tuned mechanical properties, including viscoelasticity and tensile strength. Therefore, it is not surprising that disruption of these mechanical properties is being increasingly recognized as a contributing factor to disease (Münch and Abdelilah-Seyfried 2021).

**Fig. 1:**
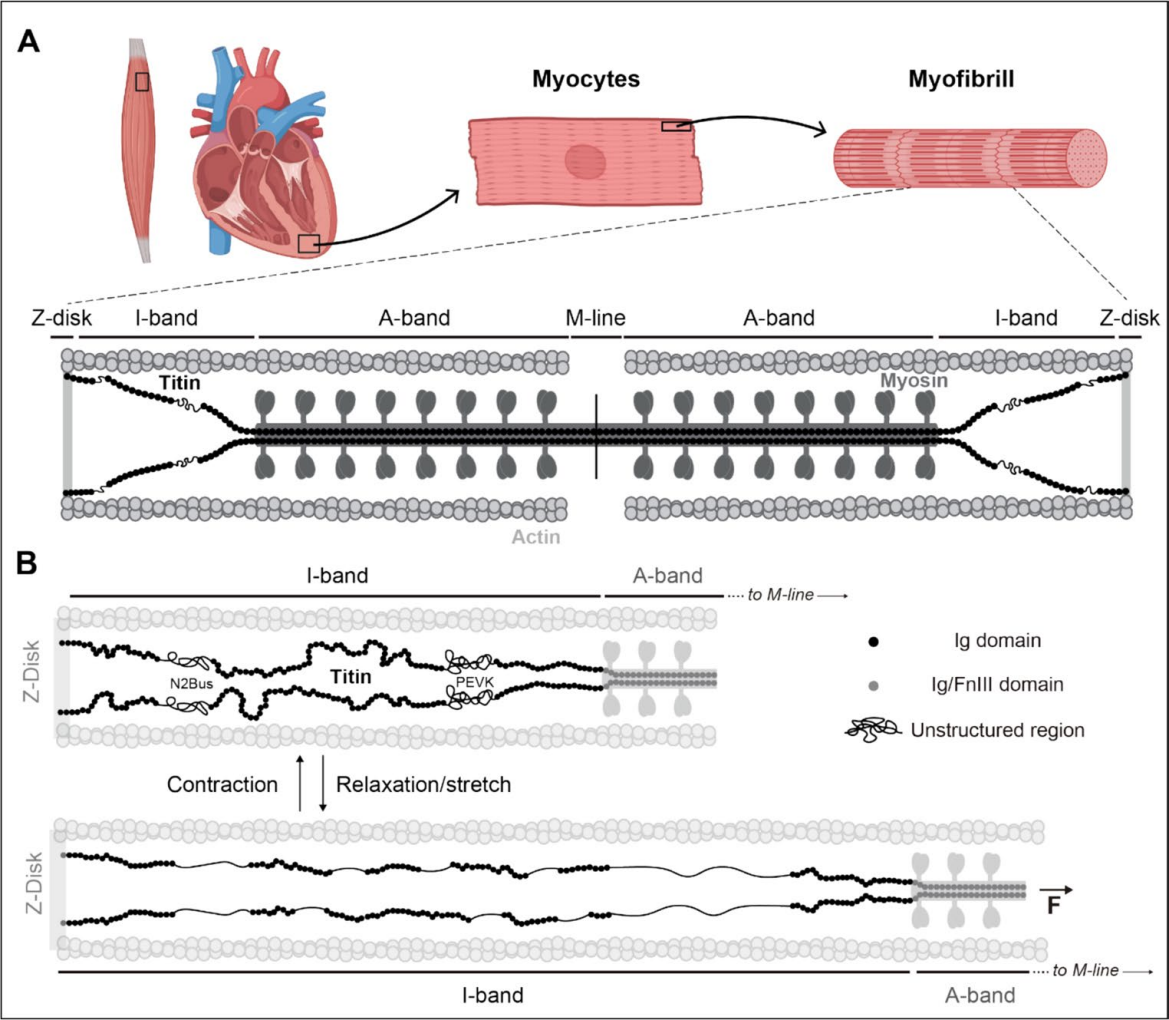
Titin structure and mechanics. **A** Schematic representation of a cardiac sarcomere and its contextualized location in muscle tissues (black boxes, not to scale). The main protein components of the sarcomere are indicated. Created with BioRender.com. **B** Schematic representation of half a sarcomere (not to scale) showing the I-band (black) and A-band (dark gray) regions of titin (N2BA isoform). Immunoglobulin-like domains (Ig), fibronectin III-like domains (FnIII), and unstructured regions are shown. During muscle relaxation, mechanical force induces the straightening of the interdomain linkers and the unstructured regions, and the unfolding of the Ig domains

Muscle organization allows for a good understanding of macroscopic tissue mechanics from the mechanical properties of cellular and subcellular components, including force-generating myofibrils, the linear association of sarcomeres (Fig. 1A). While the fundamental contribution of actomyosin filaments to active contraction is well established (Szent-Györgyi 2004), elegant recent work by Loescher et al. (2023) has confirmed that the primary source of passive forces in cardiac muscles at all levels of deformation is the cellular components of cardiomyocytes. Among these, the giant protein titin stands out as one of the most important contributors to stiffness, both in skeletal and in cardiac muscle (Y. Li et al. 2020) (Fig. 1A). In addition, titin significantly accounts for the viscous component of these forces together with other cytoskeletal components, mainly the microtubule network (Uchida et al. 2022), but also actin and intermediate filaments (Grimes et al. 2019; Loescher et al. 2023).

The mechanical properties of titin can be extensively modulated both transcriptionally and posttranslationally, providing a fine regulatory mechanism for muscle mechanics (Linke and Hamdani 2014; Loescher et al. 2022; H. L. Granzier and Labeit 2025). Likewise, pathological alteration of titin’s physical properties (e.g., mechanics and protein stability) can affect its function and lead to the development of disease, as demonstrated recently by our group (Martinez-Martin et al. 2023; López-Unzu et al., 2025 ; Silva-Rojas et al. 2025) and others (Rees et al. 2021, 2023; Freundt et al. 2025). Building on excellent previous exhaustive review papers, including Linke and Hamdani (2014) and H. L. Granzier and Labeit (2025), here we will summarize the current knowledge on titin function in the sarcomere (for less studied non-sarcomeric roles of titin, the reader is referred to (S. Labeit et al. 2006; Zastrow et al. 2006; Toffali et al. 2023)) and the mechanisms that regulate titin-based stiffness under both physiological and pathophysiological conditions. Given the strong association between titin and disease, we will provide an overview of recently described genetic variants in the titin gene (*TTN*) and their link to cardiac and musculoskeletal disease. We will also reflect on biophysical mechanisms that can contribute to pathogenicity in these genetic conditions and describe recent developments that place titin as a causative factor also in acquired cardiac disease. Finally, we will build on the current unknowns regarding the integrative functions of titin to suggest future work to better comprehend the fundamental roles of titin in health and disease, with the ultimate goal of finding effective therapies for a range of human diseases.

## Structure and mechanics of titin

Titin from vertebrates is a giant, long-lived protein of up to 4.2 MDa that extends along the whole length of half a sarcomere (Maruyama 1976; Wang et al. 1979; Bang et al. 2001; Douvdevany et al. 2024) (Fig. 1A). Structurally, human titin is composed of up to ~ 170 immunoglobulin-like (Ig) and ~ 130 fibronectin III-like (FnIII) domains arranged in series, which can be divided into different regions depending on its localization in the sarcomere. How such a large polypeptide can be integrated in sarcomeres remains controversial, although mechanisms may be different in embryonic and adult cardiomyocytes (da Silva Lopes et al. 2011; Rudolph et al. 2019; Douvdevany et al. 2024). Several independent lines of evidence demonstrate that properly tethered titin is required to stabilize sarcomeres (Radke et al. 2019; Swist et al. 2020; Pricolo et al. 2025; Silva-Rojas et al. 2025).

According to the current annotation of titin in Uniprot (accession number Q8WZ42), the N-terminal region of the protein consists of up to 7 Z-repeats and 9 Ig domains that anchor it to the Z-disk of the sarcomere (Gautel and Djinović-Carugo 2016), where titin strongly interacts with alpha-actinin (Grison et al. 2017) and telethonin (Bertz et al. 2009). On the opposite side of the protein, the C-terminal region is integrated into the M-band, although recent structural data on intact sarcomeres suggest that only half of the titin molecules reach this region (Tamborrini et al. 2023). When present, the M-band region of titin comprises the titin kinase domain, a pseudokinase suggested to participate in mechanosensing and degradation mechanisms (Lange et al. 2005; Puchner et al. 2008; Bogomolovas et al. 2014, 2021; Tamborrini et al. 2023), and 10 Ig domains that interact with myomesin and obscurin (Fukuzawa et al. 2008; Pernigo et al. 2010), among others (Gautel and Djinović-Carugo 2016). The I-band part of titin is the most variable region of the protein, since it is heavily affected by alternative splicing (Savarese et al. 2018). This region consists of several Ig domains and mostly unstructured segments like the PEVK and the N2Bus stretches, which can respond to intracellular calcium levels (D. Labeit et al. 2003). Interestingly, recent work has identified a folded and mechanically weak globular structure within the cardiac-specific N2Bus region (Sun et al. 2024). Finally, the A-band of titin is composed of super-repeats of Ig and FnIII domains whose periodicity matches that of the myosin crowns (S. Labeit et al. 1992; Dutta et al. 2023; Tamborrini et al. 2023). Because of this A-band arrangement, titin has been proposed as a ruler for thick filament assembly (H. Granzier et al. 2014; Linke and Hamdani 2014; Tskhovrebova et al. 2015; Tonino et al. 2017, 2019; Bennett et al. 2020; Linke 2023).

The mechanical function of titin resides mainly in the I-band, which is therefore considered the mechanically active region of the protein (Linke et al. 1998; Linke and Hamdani 2014). When a force is applied to titin, the interdomain linkers and the unstructured regions of the protein stretch, providing entropic elasticity to the sarcomere (H. Li et al. 2002; Von Castelmur et al. 2008; Lanzicher et al. 2020). At increased sarcomere lengths, the forces experienced by titin molecules also induce the unfolding of Ig domains in the I-band of titin (M. Kellermayer et al. 1997; Rief et al. 1997; Tskhovrebova et al. 1997), a process that can be particularly relevant at physiological temperatures (Yu et al. 2021). Termed the sequential titin-extension hypothesis, the series of extension and unfolding events sets titin as a molecular spring driven primarily by entropic elasticity yet also featuring enthalpic components that contribute to its viscoelastic behavior (Minajeva et al. 2001; H. Li et al. 2002; Linke 2023). Once the force ceases, the elastic regions recoil elastically whereas the Ig domains can regain their folded structure (Fig. 1B). The mechanical energy released during these events has been proposed to contribute to the early phase of sarcomere active shortening (Opitz et al. 2003). Indeed, recent studies have shown that domain refolding generates mechanical work that can act cooperatively with myosin motors to generate force (Rivas-Pardo et al. 2016, 2020; Mártonfalvi et al. 2017; Eckels et al. 2019). According to these observations, titin may not only play a role in the passive forces of muscle but would also contribute to active contraction (Eckels et al. 2018). Titin mechanics has also been proposed to contribute to the Frank-Starling law of the heart (Ait-Mou et al. 2016), potentially through the modulation of both thick and thin filament structures, as well as to residual force enhancement in myofibers (Hessel et al. 2024). Furthermore, force transmission across titin is required for the stability of the A-band in contracting sarcomeres (Y. Li et al. 2020). It has also been proposed that interactions between the thin filament and titin can modulate the mechanical properties of the I-band of the protein, impacting the overall stiffness of cardiomyocytes particularly during active contraction (Linke et al. 2002; Squarci et al. 2023; Desai et al. 2025).

## Regulation of titin-based stiffness

**Fig. 2:**
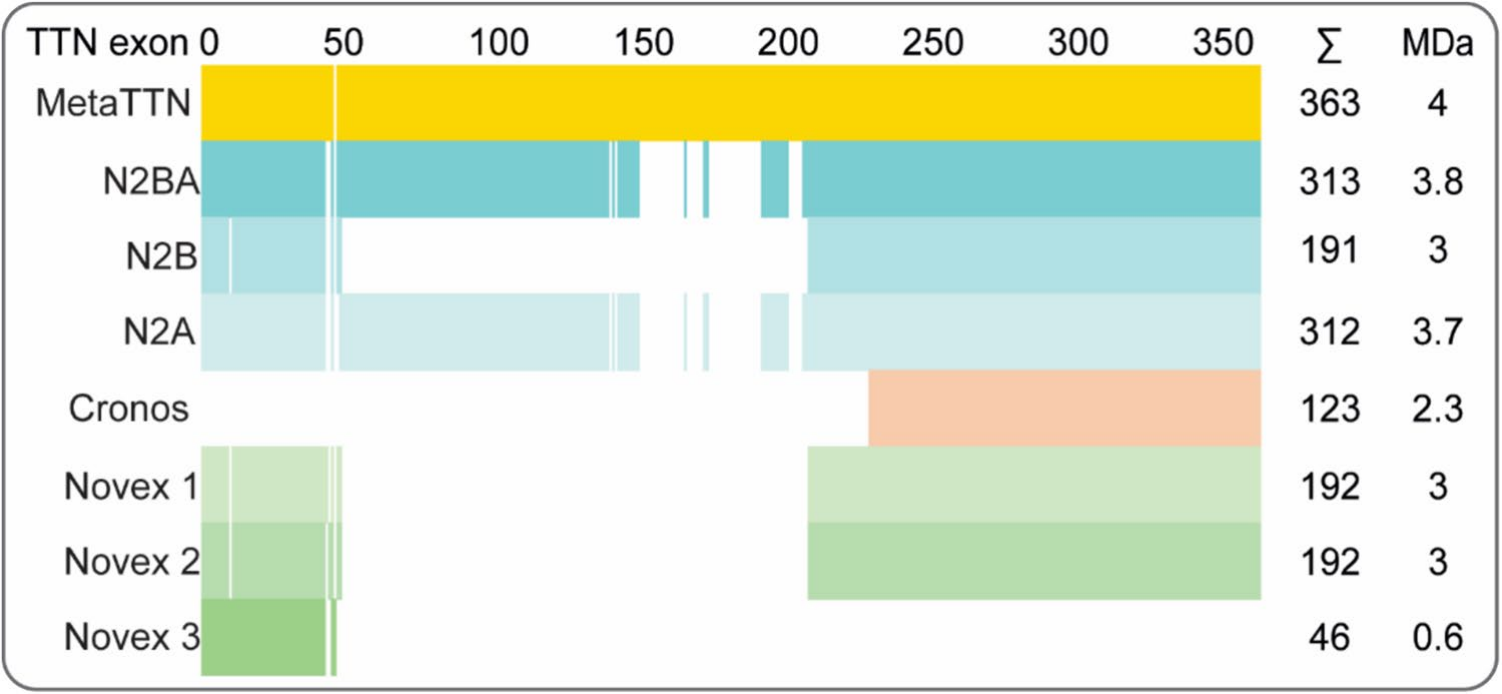
Exon inclusion in the different titin isoforms described in adult human muscles. Please note that there are exons in the metatranscript of titin that are not expressed in any of the detected isoforms. Total size of isoforms in terms of number of included exons and size of the isoform are indicated. Adapted from https://www.cardiodb.org/titin/ and Zaunbrecher et al. (2019)

The mechanical properties of titin can be finely tuned by a variety of biological processes. The best-established mechanism is the alternative splicing of titin mRNA, which mainly affects the I-band region of the protein, generating several isoforms of different lengths and domain composition. Titin is encoded by the *TTN* gene, which contains 363 and 347 exons in humans (ENST00000589042.5) and in mice (ENSMUST00000099981.10), respectively. Alternative splicing of the titin mRNA or usage of an alternative promoter in the case of the Cronos isoform yields the different titin isoforms expressed in muscles (Fig. 2). The main cardiac isoforms are N2BA, N2B, Cronos, and Novex 1–3, while skeletal muscles express mostly N2A isoforms lacking the N2B segments and Cronos. Cronos and Novex are suggested to have rather structural and regulatory roles that might be important for sarcomere assembly but have low relevance in the mechanics of titin (Bang et al. 2001; Zou et al. 2015; D. Kellermayer et al. 2017; Zaunbrecher et al. 2019).

**Fig. 3:**
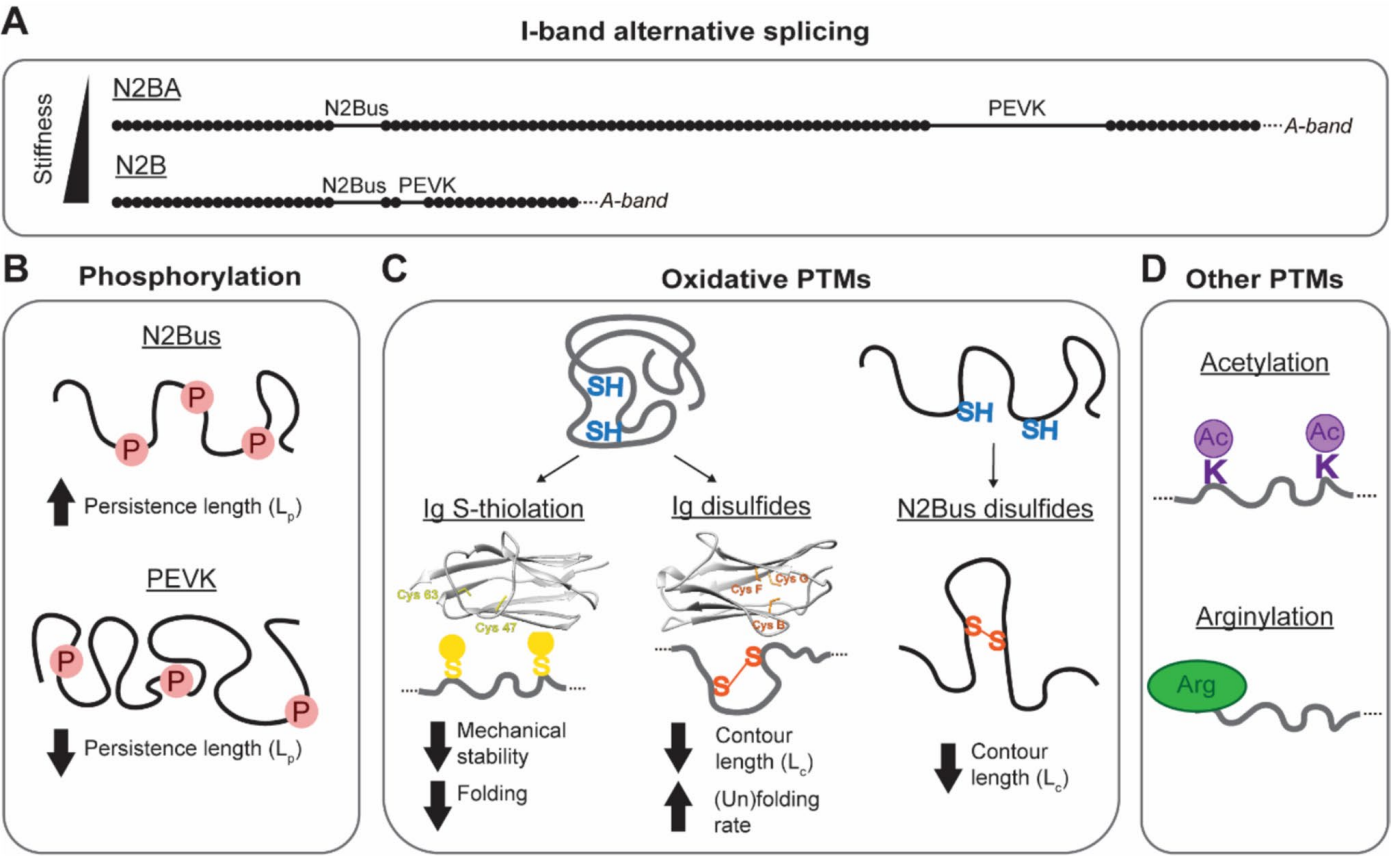
Regulation of titin-based stiffness. **A** Domain architecture of the I-band region of the main titin isoforms in cardiac muscle. Ig domains are represented as black circles and unstructured regions as black lines. The ratio between the compliant N2BA and the stiffer N2B isoforms modulates titin-based stiffness. **B** Phosphorylation (represented as pink circles) of the unstructured regions of titin modulates titin mechanical properties. Phosphorylation of the N2Bus increases titin *L*_p_ while phosphorylation of the PEVK reduces it. **C** Oxidation of titin cysteines in Ig domains (left and middle) and N2Bus (right) modulates titin mechanics. S-thiolation (yellow circles) of titin Ig domains decreases the mechanical stability of titin domains and their folding, while disulfide formation (orange lines) reduces the contour length (Lc) of the targeted regions but increases the (un)folding rates of titin domains. Reduced cysteines are shown in blue. Cartoon representations of protein domains indicate the position of the structurally conserved cysteines in titin that have been shown to be targets of S-thiolation (left, yellow, represented in the structure of domain I27 (PDB ID: 1TIT)) or disulfide bonds (middle, orange, represented in the structure of domain I74 (homology model of positions 9079–9168 of Uniprot Q8WZ42 entry)). **D** Acetylation (top, purple) and arginylation (bottom, green) of titin also target titin molecules, although their mechanical effects are less studied

The contribution of titin splicing to muscle physiology has been extensively demonstrated, at very least at the correlation level. Differences in titin isoform content are seen between species and even between heart compartments within the same species, correlating with myofibrillar passive stiffness (Cazorla et al. 2000; Neagoe et al. 2003; Lahmers et al. 2004). The isoform profile, including splice variants of the N2BA and N2A isoforms, also changes dramatically during heart and skeletal muscle development (Opitz et al. 2004; Warren et al. 2004; Opitz and Linke 2006; Ottenheijm et al. 2009). In the heart, the ratio between the longer and compliant N2BA isoform and the shorter and stiffer N2B correlates with the ventricular stiffness (Freiburg et al. 2000) (Fig. 3A). The switch between these two isoforms is controlled, at least partially, by the RNA binding motif protein 20 (RBM20) (Guo et al. 2012), which promotes the expression of the N2B isoform by repressing the splicing-in of I-band exons (S. Li et al. 2013). The mechanisms that regulate titin isoform switching are still not completely understood, although biochemical pathways including those elicited by insulin and thyroid hormone 3 have been shown to affect RBM20 activity (C. Zhu et al. 2015, 2017). Remarkably, the N2BA:N2B ratio has been observed to increase in cases of disease such as ischemia (Neagoe et al. 2002), dilated cardiomyopathy (DCM) (Makarenko et al. 2004; Nagueh et al. 2004) and in Marfan syndrome (D. Kellermayer et al. 2025). In contrast, the N2BA:N2B ratio decreases during volume overload of the heart, at least in experimental mice (Hutchinson et al. 2015).

Regulation of titin mechanics can also be achieved by posttranslational modifications (PTMs), which are potent regulators of protein nanomechanics (Alegre-Cebollada 2021). The best-studied modification in terms of titin stiffness modulation is the phosphorylation of the unstructured regions N2Bus and PEVK. Phosphorylation of the N2Bus segment by protein kinase A (PKA) (Yamasaki et al. 2002), protein kinase G (PKG) (Krüger et al. 2009), calcium/calmodulin-dependent protein kinase II delta (CaMKIIδ), or extracellular signal-regulated kinase 2 (ERK2) (Hamdani et al. 2013b; Perkin et al. 2015) has been shown to increase the persistence length (*L*_p_) of titin, correlating with reduced cardiac stiffness. In contrast, phosphorylation of the PEVK region of titin by protein kinase C (PKC) decreases *L*_p_ and induces increased titin-based passive force (Hidalgo et al. 2009; Anderson et al. 2010) (Fig. 3B). Notably, as further discussed below, altered titin phosphorylation is commonly found in cardiac (Koser et al. 2019) and musculoskeletal (Ottenheijm et al. 2012) disease, but can also be the result of physiological adaptation (Müller et al. 2014). From a mechanistic point of view, it remains unclear how phosphorylation of a few sites can have such dramatic effects on the *L*_p_ of long polypeptides, and more so on the mechanics and structure of the whole titin filament (Balogh-Molnár et al. 2025). In this regard, the potential role of Ig domain phosphorylation has not been addressed so far even though multiple potentially relevant phospho-sites at Ig domains have been detected (Hamdani et al. 2017). Also, it is possible that mechanical effects downstream of phosphorylation are contributed by modulation of protein-protein interactions (Raskin et al. 2012).

A more recently studied mechanism for the modulation of titin-based stiffness is the modification of the protein by redox PTMs (Herrero-Galán et al. 2019). The elastic I-band of titin contains > 100 cysteine residues that can be targeted by oxidative modifications. Although these cysteines are generally cryptic, they can be exposed and modified upon unfolding of the domain (Alegre-Cebollada et al. 2014). In fact, recent work has shown that a fraction of them can be found as oxidized in titin molecules in vivo either in physiological (Kramer et al. 2018; Herrero-Galán et al. 2022) or in pathophysiological conditions (Loescher et al. 2020). The location of the cysteines within the fold of titin domains determines the modifications that can target them. Many titin domains contain cysteines that are clustered together at distances and orientations that enable formation of disulfide bonds (Mayans et al. 2001; Giganti et al. 2018). Other domains contain cysteines that are too distant from each other to be crosslinked by disulfide bonds but can be modified by alternative non-crosslinking modifications such as S-glutathionylation (Alegre-Cebollada et al. 2014) (Fig. 3C). The distinction between disulfide bonds and other modifications is fundamental, as they have different effects on titin stiffness. S-glutathionylation of cryptic cysteines in titin Ig domains increases titin compliance by reducing the mechanical stability of the domains and by impairing domain folding (Alegre-Cebollada et al. 2014). In contrast, the formation of disulfide bonds between cysteines belonging to a triad of structurally conserved cysteines increases titin stiffness by reducing the contour length of the unfolded domain (Giganti et al. 2018). Disulfide bonds also accelerate both unfolding and refolding of titin domains, with opposing effects on overall stiffness (Giganti et al. 2018; Eckels et al. 2019). To add further complexity, the unstructured region N2Bus can also be crosslinked by disulfide bonds, which increases titin stiffness (Grützner et al. 2009). Monte Carlo–based computer simulations predict that the overall effect of disulfide bonds is to stiffen titin, although at low forces and for long titin isoforms, they could result in softening through the induction of protein unfolding (Herrero-Galán et al. 2022) (Fig. 3C).

Titin residues can also be targeted by other PTMs such as acetylation (Abdellatif et al. 2021; Koser et al. 2022) or arginylation (Leite et al. 2016), both resulting in stiffening of muscle tissue. It is important to note that while phosphorylation, oxidation, and acetylation have been detected in the mechanically active I-band region of titin, arginylation is only detected in the A-band, and its stiffening effect has been suggested to originate from changes in protein-protein interactions rather than from the alteration of the elastic properties of titin (Fig. 3D).

## The role of titin in mechanosignaling

Beyond the mechanostructural roles discussed above, emerging evidence places titin as a central hub for mechanosensing in (cardio)myocytes (Rudolph et al. 2020). For instance, the protein CCDC141 has been identified as an interactor of A-band titin that also binds to nesprin-1, adapting cardiomyocytes to mechanical stress (Hanashima et al. 2025). However, a direct role of titin-based mechanosignaling in the function of CCDC141 remains to be demonstrated. In addition, a titin-based force-feedback system described in *Drosophila* has been proposed to regulate the length of the thick filament, although the underlying molecular mechanisms remain incompletely understood (Loreau et al. 2025).

At the molecular level, distinct regions of titin can act as hubs for mechanosensing and mechanotransduction, allowing muscle cells to respond to mechanical stress. At the Z-disk, titin participates in a mechanosensory complex with MLP and T-cap (Knöll et al. 2002). At the I-band region, force-dependent modulation of protein-protein interactions involving titin can contribute to mechanosensing and mechanotransduction, although experimental validation remains scarce. A notable exception is the demonstration that the binding affinity between FHL2 and the N2Bus region of titin is activated by force (Sun et al. 2024), which can be important for mechanotransduction during volume overload of the heart (Strom et al. 2024). Other titin interactors have been suggested to participate in titin-mediated mechanosensing. For instance, MARP proteins have been shown to regulate passive force by “locking” titin to thin filaments, potentially protecting myofibrils from damage under excessive stretch (R. J. van der Pijl et al. 2021; Zhou et al. 2021). Interestingly, MARPs proteins limit longitudinal hypertrophy in a unilateral diaphragm denervation model used to study titin-stiffness-dependent muscle hypertrophy (R. van der Pijl et al. 2018, 2025). Similarly, a titin/FHL-1 complex has been proposed to mediate response to mechanical stress in cardiomyocytes (Sheikh et al. 2008). Furthermore, molecular chaperones such as αB-crystallin and Hsp27 bind titin under stress conditions (Golenhofen et al. 2002; Bullard et al. 2004), limiting the extension of unstructured regions and Ig domains (Y. Zhu et al. 2009) and preventing aggregation-induced stiffening (Kötter et al. 2014). Finally, the M-line region has also been implicated in mechanosignaling via its pseudokinase domain. Mechanical stress induces conformational changes within this domain, enabling its ubiquitination by MuRF1 and MuRF2 (Puchner et al. 2008; Bogomolovas et al. 2014). This modification leads to the recruitment of Nbr1 and p62, receptors involved in autophagy and sarcomere turnover (Bogomolovas et al. 2021).

## Titin in genetic disease

The importance of titin for striated muscle physiology is further supported by the association of the protein with disease. Indeed, variants in the *TTN* gene cause multiple forms of skeletal (Oates et al. 2018; Savarese et al. 2020; Coppens et al. 2025) and cardiac disease (LeWinter and Granzier 2014; Weston et al. 2024). While the development of skeletal muscle disease requires that pathogenic *TTN* variants are present in homozygosis, compound heterozygosis (Rees et al. 2021) or in combination with pathogenic variants in other genes (Töpf et al. 2024), variants associated with cardiomyopathies exhibit a dominant phenotype and are pathogenic already in heterozygosis (Gerull et al. 2002; Hastings et al. 2016; Domínguez et al. 2023). These heart-disease-causing *TTN* variants are most probably incompatible with life when present in homozygosis or compound heterozygosity (Radke et al. 2019; M. wei Li et al. 2024).

DCM is the best-known example of a genetic disease caused by variants in titin; in most cases, DCM-causing variants generate premature stop codons (i.e., truncating titin variants or *TTN*tvs) (Herman et al. 2012). Besides DCM, *TTN*tvs have also been associated with peripartum cardiomyopathy (Van Spaendonck-Zwarts et al. 2014; Ware et al. 2016; Spracklen et al. 2021), primary myocardial fibrosis (Junttila et al. 2018), atrial fibrillation (Ahlberg et al. 2018; Choi et al. 2018), chemotherapy-induced cardiomyopathy (Garcia-Pavia et al. 2019), and with several muscle pathologies that often involve cardiac phenotypes (Rees et al. 2021). Indeed, the presence of *TTN*tvs has also been shown to increase the risk of several different cardiac conditions, generally in combination with other factors (Shetty et al. 2024a). For example, *TTN*tvs have been shown to favor cardiac remodeling (Schafer et al. 2017) and to be associated with a higher risk of arrhythmias and heart failure (Haggerty et al. 2019; Shetty et al. 2024b). In addition, DCM patients carrying *TTN*tvs in heterozygosis can also have mild skeletal muscle involvement (Skriver et al. 2023). It remains unknown why seemingly equivalent *TTN*tvs can lead to different cardiac conditions. In this regard, a recent report has proposed that the proportion of non-truncating transcripts *TTN*tvs may influence whether carriers develop cardiac conduction disease or DCM (Ishikawa et al. 2025).

Missense variants in titin, which result in single amino acid substitutions, can also contribute to skeletal muscle and heart disease. For instance, pathogenic titin missense variants have been reported in cases of DCM (Gerull et al. 2002; Itoh-Satoh et al. 2002; Hastings et al. 2016; Akinrinade et al. 2019; Meurs et al. 2019; Domínguez et al. 2023), skeletal muscle disease (Hedberg et al. 2014; Rees et al. 2021), arrhythmogenic cardiomyopathy (ACM) (Taylor et al. 2011), restrictive cardiomyopathy (Peled et al. 2014), and atrial fibrillation (Pavel et al. 2025). Missense variants in titin have also been linked to the development of hypertrophic cardiomyopathy (HCM) (Satoh et al. 1999); however, the association of titin with HCM remains controversial (Weston et al. 2024).

In the following sub-sections, we further expand on the available information on the well-established causative role of *TTN* variants in dilated cardiomyopathy, as well as on the emerging role played by *TTN* variants in musculoskeletal diseases.

### Titin variants in dilated cardiomyopathy

Among the various heart diseases related to titin, DCM stands out because of its strong association with *TTN* variants. DCM is characterized by left ventricle dilatation and reduced ventricle contractility potentially leading to serious complications such as heart failure and arrhythmias. Indeed, DCM is the most common indication for heart transplantation (Weintraub et al. 2017; Reichart et al. 2019; Schultheiss et al. 2019). Early diagnosis of the disease is important for optimal patient care, but it is hindered by the asymptomatic nature of the early stages of the disease (Japp et al. 2016).

Pathogenic variants linked to DCM have been detected in > 30 genes, including those targeting sarcomere proteins (Reichart et al. 2019). Among them, *TTN*tvs account for ~ 25% of the familial and ~ 18% of the sporadic cases of the disease, making *TTN*tvs the most common cause of DCM (Herman et al. 2012). Most DCM-associated *TTN*tvs originate from variants located in the A-band region of the protein followed by counterparts in the constitutively expressed exons of the I-band (Herman et al. 2012; Schafer et al. 2017). In contrast, *TTN*tvs found in healthy individuals are randomly distributed throughout the titin sequence (Schafer et al. 2017). Titin’s extensive alternative splicing likely contributes to this site-dependent effect, as mutations in spliced-out regions may impact less on cardiomyocyte fitness. Another proposed explanation for the variable clinical severity of *TTN*tvs is that the expression of the C-terminal titin isoform Cronos could potentially compensate for truncations in the I-band region, but not for A-band variants (Zou et al. 2015). Finally, it is also possible that inefficient translation termination of certain *TTN*tvs contributes to explaining the absence of pathological consequences in specific cases (van Heesch et al. 2019).

At the protein level, pathomechanisms induced by *TTN*tv appear to stem both from reduced full-length titin levels (haploinsufficiency) and from new toxic properties in truncated titin peptides (Fomin et al. 2021; McAfee et al. 2023), which eventually cause sarcomere deficiency (Hinson et al. 2015). These truncated titin molecules have been suggested to form aggregates that can disrupt the protein quality control system of the cell (Fomin et al. 2021) and to induce sarcomere disarrangement affecting sarcomere organization and mechanosensing (McAfee et al. 2023; D. Kellermayer et al. 2024). In this regard, truncated titin molecules can bear load, but their attachment to the A-band of sarcomeres is less stable, causing recoiling of the protein towards the Z-disk under stress (McAfee et al. 2023). A similar recoiling has been observed when titin cleavage is induced in skeletal muscle (Silva-Rojas et al. 2025). Interestingly, titin cleavage in cardiac muscle induces a fast reactive fibrotic response, suggesting that fibrosis typically found in *TTN*tv-induced DCM is contributed by the toxicity of unloaded titin molecules (López-Unzu et al. 2025). Nevertheless, the fact that increasing both full-length and truncated titin levels leads to improved contractile function of engineered heart tissues carrying *TTN*tvs suggests that titin haploinsufficiency may be more relevant than truncated titins for the pathophysiology of the disease (Ghahremani et al. 2024). It will be interesting to test though whether this scenario also holds in models of disease that can recapitulate DCM-associated fibrosis. Considering the pathogenicity associated with titin haploinsufficiency, a gene variant in a titin intronic enhancer has been linked to DCM (Kim et al. 2024).

Missense variants in titin are also frequently observed in DCM patients (Herman et al. 2012; Akinrinade et al. 2019), but determining whether they are pathogenic or not is complicated by the fact that this class of variants as a whole is also found in the general population at a frequency that is not compatible with DCM prevalence (Begay et al. 2015). As a result, most titin missense variants remain classified as benign or as variants of uncertain significance (VUS) (Ware and Cook 2018; Akinrinade et al. 2019; Morales et al. 2020). To date, only three titin missense variants have been proposed to cause DCM based on strong cosegregation with disease: p.A178D (Hastings et al. 2016; H. Jiang et al. 2021), p.W976R (Gerull et al. 2002; Hinson et al. 2015), and p.C3575S (Domínguez et al. 2023). The pathogenic mechanisms of missense variants may be more variable than those proposed for *TTN*tv, since they can affect any of the > 30,000 residues of the protein. On that note, p.A178D disturbs the interaction between titin and telethonin (Hastings et al. 2016), which accumulates and initiates a proteo-toxic response (H. Jiang et al. 2021), while p.W976R and p.C3575S result in destabilization of the hydrophobic core of the parent domain that correlates with reduced contractility of affected cardiomyocytes (Gerull et al. 2002; Hinson et al. 2015; Domínguez et al. 2023; Martinez-Martin et al. 2023). Remarkably, missense titin variants destabilizing the hydrophobic core of titin domains are specifically enriched in DCM patients for which no other genetic cause has been identified (Martinez-Martin et al. 2023). How destabilization of a single titin domain out of hundreds causes DCM remains unknown.

### Titin variants in skeletal myopathies

Together with *DMD* and *RYR1*, *TTN* is among the most commonly mutated genes in myopathies (Savarese et al. 2016; Dowling et al. 2021). Myopathies can be classified according to their disease onset, affected muscles, and histopathological hallmarks. For instance, muscle dystrophies are characterized by membrane fragility and myofiber death, while congenital myopathies generally show structural abnormalities. Remarkably, variants in the *TTN* gene cause both muscle dystrophies and congenital myopathies and the more general term “titinopathies” has been coined to encompass all muscle conditions resulting from mutations in titin, which typically need to occur in homozygosis or compound heterozygosis for disease to develop (Table 1). While *TTN*tvs have historically been the primary focus for geneticists interested in inherited muscle disease, missense variants can also be pathogenic (Rees et al. 2021), which has opened a *Pandora’s box* in titin genetics. As for DCM, further developments are needed to fully apprehend the pathogenicity potential of missense variants targeting titin. Similarly, how specific titin variants drive the different titinopathies remains incompletely understood. As discussed below, generation of animal models of titinopathies could be highly informative in this regard.

**Table 1:** Features of skeletal muscle diseases caused by *TTN* variants. *NR*, not reported

Condition feature	TMD/LGMD	EDMD	CNM/MmD-HD	HMERF	AMC
Onset	Adult	Young	Adult	Young	Prenatal
Region	C terminal	C terminal	All through	C terminal	Metatranscript exons
Muscles affected	Anterior part of lower legs	Upper and lower limb muscles and stiff joints	Cranial, axial, proximal, and distal muscles. Joint contractures and cardiac involvement	Upper and lower limb and respiratory muscles	Generalized hypotonia and contractures
Fiber size variability	Yes	Yes	Yes	Yes	Yes
Central nuclei	Yes	Yes	Yes	Yes	Yes
Fibrosis	Yes	Yes	Yes	Yes	Yes
Cytoplasmic bodies	Yes	Yes	NR	Yes	Yes
Vacuoles	Yes	Yes	No	Yes	No
Stiff joints/contractures	Yes	Yes	Yes	Yes	Yes

Among the dystrophic titinopathies, the less severe cases are tibial muscle dystrophy (TMD) and limb girdle muscle dystrophy (LGMD R10 titin-related) (Udd et al. 1991, 2005; Partanen et al. 1994; Hackman et al. 2002). These conditions share a clear hotspot in the C-terminal zone of titin near the M-line, where truncating variants or in-frame deletions enhance the predisposition to cleavage by ubiquitous calpains (Charton et al. 2015). Similar mutations also cause Emery-Dreifuss-like muscular dystrophy (EDMD) phenotypes without cardiomyopathy, where joints are also affected (De Cid et al. 2015). In all these cases, the affected titin molecules are not expected to reach the M-line, likely compromising sarcomere stability. This instability could be behind the various forms of protein aggregation observed by electron microscopy, including cores or disarrays.

Congenital titinopathies caused by titin mutations include centronuclear myopathy (CNM) and autosomal recessive multi-minicore disease with heart disease (MmD-HD) (Ceyhan-Birsoy et al. 2013; Chauveau et al. 2014; Palmio et al. 2019). Both conditions are caused by homozygous or compound heterozygous mutations in *TTN* that introduce premature stop codons at different regions of the protein, with predicted consequences on titin anchoring to both ends of the sarcomere. Cores in electron micrographs also appear in this context and, given the predicted effect of mutations on titin, they presumably arise from sarcomere breakdown due to defective titin scaffolding (Ávila-Polo et al. 2018). Sarcomeric disarrays are also observed in patients with C-terminal variants in *TTN* that are associated with hereditary myopathy with early respiratory failure (HMERF) (Chinnery et al. 2001; Pfeffer et al. 2012; Hedberg et al. 2014).

**Fig. 4:**
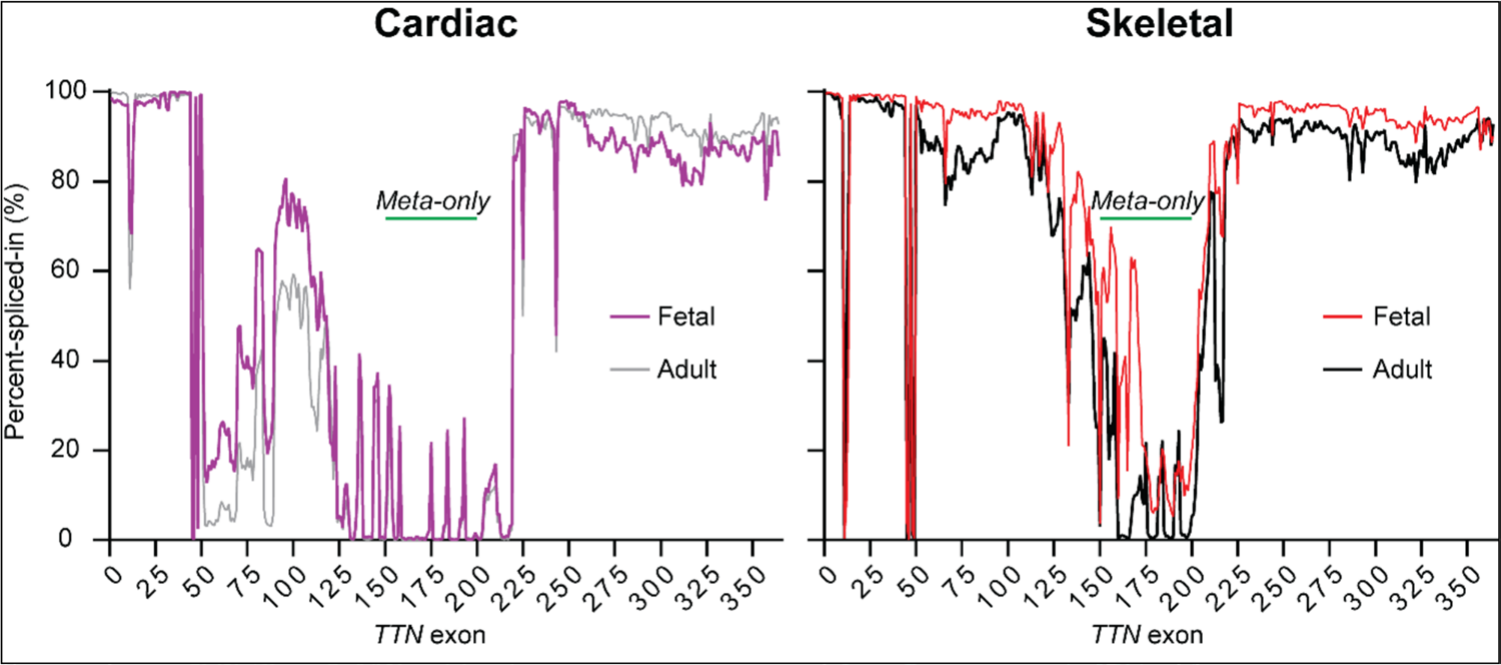
Expression of *TTN* exons in adult and fetal muscles. Bulk RNAseq analysis of fetal and adult tissues reveals differences in the percent-spliced-in values of *TTN* exons. Cardiac samples show lower expression of exons 50–150 than skeletal muscles. This region corresponds to PEVK-expressing exons, which are incorporated into the N2BA cardiac isoform (representing 30–40% of the titin molecules expressed in cardiac tissue (Neagoe et al. 2002)) and the N2A skeletal isoform. Metatranscript-only *TTN* exons correspond to exons 158–201 (labelled as “Meta-only”), with some exons only expressed in fetal skeletal muscle. The represented data are from Di Feo et al. (2024).

Another condition recently linked to titin mutations is arthrogryposis multiplex congenita (AMC) (Fernández-Marmiesse et al. 2017; Oates et al. 2018; Bryen et al. 2020; Di Feo et al. 2024), a disorder characterized by prenatal-onset of muscle hypotonia and joint contractures (arthrogryposis). AMC patients typically exhibit hypotonia from prenatal stages, and the disease course depends on the usage of the mutated exons. Notably, cardiac defects are not commonly reported (Di Feo et al. 2024). Most of the AMC-causative mutations are autosomal recessive truncating variants in homozygosity or compound heterozygosity affecting at least one metatranscript-only exon (i.e., exons 158–201) (Pérez‐Vidarte et al. 2025). In Fig. 4, we show percent-spliced-in (PSI) values of *TTN* exons, which shows that expression of metatitin exons is almost absent in adult tissues compared to fetal counterparts. The highest expression of metatitin exons is found in fetal skeletal muscle, which may explain the absence of cardiac defects in AMC-*TTN* patients (Di Feo et al. 2024).

### Current challenges in the clinical interpretation of titin variants

As discussed above, the central role of titin in striated muscle physiology is reflected in the strong association of the protein with muscle and heart disease (LeWinter and Granzier 2014; Ahlberg et al. 2018; Choi et al. 2018; Weston et al. 2024). However, the presence of titin variants in healthy individuals complicates the evaluation of the pathogenicity of newly discovered variants. Even *TTN*tvs, which are typically categorized as pathogenic, are detected in 0.5–1% of the general population, a frequency significantly higher than the prevalence of DCM (Norton et al. 2013; Walsh et al. 2017; Bourfiss et al. 2022). This situation is even worse for missense variants. Indeed, most titin missense variants identified in patients are classified as VUS, a genetic category that has little or no use in the clinic (Richards et al. 2015). This situation emphasizes the need for thorough validation when associating variants with disease (Herman et al. 2012; Roberts et al. 2015; Ware and Cook 2018).

The initial approach to assessing the pathogenicity of a newly discovered variant relies on genetic criteria. For instance, variants prevalent in the general population are less likely to be pathogenic, whereas those with low allele frequencies are more likely to be disease-causing. However, for a protein the size of titin, it is not uncommon to find rare missense variants in healthy individuals (Laddach et al. 2017; Weston et al. 2025). Hence, genetic studies aim for cosegregation analysis of the variant of interest with the disease in affected families (Hastings et al. 2016; Domínguez et al. 2023), an approach that is typically inconclusive due to the limited number of relatives and the incomplete penetrance of the disease. Alternatively, geneticists can look for variant enrichment in large cohorts of patients (Herman et al. 2012).

Considering the scarcity of detailed genetics data for many variants, especially missense ones, functional analysis of their damaging effect appears as useful complementary information in pathogenicity assessment (Richards et al. 2015). Classical in vitro methods have attempted to assess changes in ligand binding (Satoh et al. 1999; Rudloff et al. 2015; Hastings et al. 2016), protein structure (Bogomolovas et al. 2016), protein stability (Rudloff et al. 2015; Rees et al. 2021; Domínguez et al. 2023), or mechanical properties (Anderson et al. 2013; Zuo et al. 2021) resulting from titin variants. The main limitation of these methods is that full-length titin cannot be recombinantly expressed due to its size and complexity, and experiments are based on single domains or short fragments of the molecule, which can hinder the characterization of pathogenicity mechanisms. As an alternative, cellular models incorporating titin variants can be used. Although 2D cell cultures of human induced pluripotent stem cells (hiPSC)–derived cardiomyocytes can provide useful information to address structural and functional defects resulting from titin alterations (Fomin et al. 2021; Domínguez et al. 2023), generation of microtissue structures might be needed to fully uncover the pathogenic effects of the variant (Hinson et al. 2015). Functional studies can also be based on animal models (most typically fish and mice) engineered to carry the genetic variant of interest (Marcello et al. 2022). One of the challenges of this approach is that the phenotype developed by the animals does not always resemble the one observed in humans. It is not uncommon that variants causing disease in human patients in heterozygosis require expression in homozygosis in animal models to show phenotypes (H. Jiang et al. 2021), or that even in this case, they exhibit only mild phenotypes (Bogomolovas et al. 2016). Sometimes this challenge can be overcome by stressing the mutant animals, which may show increased sensitivity to the stressor (H. Jiang et al. 2021).

It should be noted that the fact that titin variants have been identified in different pathologies, including cardiac and skeletal conditions, suggests that the disease phenotype is influenced by mutation-dependent downstream mechanisms. For example, variants targeting Ig or FnIII domains may have different consequences. Similarly, a missense variant affecting exposed regions is more likely to perturb biomolecular interactions (Hastings et al. 2016), whereas another one disrupting the domain hydrophobic core probably results in reduced protein stability (Gerull et al. 2002; Rees et al. 2021; Domínguez et al. 2023; Martinez-Martin et al. 2023). Another obvious consequence of variant localization is its inclusion in specific isoforms. For example, cardiac and skeletal muscle express different isoforms including tissue-specific exons (Laddach et al. 2017) (Fig. 4), which can explain why a given pathogenic variant affects or not specific muscles. Furthermore, recent discovery of AMC-related *TTN* has demonstrated the relevance of prenatal titin isoforms and exon PSI during variant pathogenicity assessment (Di Feo et al. 2024; Vatta et al. 2025). Another factor that needs to be considered is the biochemical environment of the specific region targeted by a given variant. This can explain why very similar variants can result in different phenotypes. For instance, *TTN*-p.A178D has been shown to impair binding to telethonin leading to DCM and left ventricular non compaction (Hastings et al. 2016), while the equivalent variant in an adjacent domain (p.A82D) causes congenital fiber type disproportion with no cardiac involvement (Rees et al. 2021; Weston et al. 2024). Finally, characterizing the effects of titin variants is further complicated by the fact that their associated phenotype may require digenic inheritance with variants targeting other genes (Töpf et al. 2024) or a “second hit” such as pregnancy (Restrepo-Córdoba et al. 2024) or alcoholism, as it has been observed with some *TTN*tv (Ware et al. 2018).

## Titin in acquired disease

Recent advances in genetic studies and genetically engineered models have enabled a better understanding of titin biology, firmly establishing the protein as a major genetic determinant of cardiac and muscle disease. In contrast, the role of titin in acquired disease remains less clear, although growing evidence suggests an important contribution of titin in pathological remodeling, particularly in the heart.

The mechanisms linking titin to acquired disease are diverse and not completely understood. For example, dysregulation of the mechanisms that control titin-based stiffness may be a cause of disease. As described above, alterations in titin isoform ratios are present in many forms of cardiac pathology, although it remains unclear whether they serve as a compensatory mechanism for the changes in myocardial stiffness or whether they contribute directly to disease progression. In this context, it is interesting that pathogenic variants targeting RBM20 have been linked to DCM, suggesting a causative role of alterations of titin splicing in disease (Brauch et al. 2009; D. Li et al. 2010; Beqqali et al. 2016). Changes in titin PTMs have also been associated with several diseases (Loescher et al. 2022). Hyperphosphorylation of the PEVK segment and hypophosphorylation of the N2Bus region have been detected in heart failure, diabetes, and diastolic dysfunction correlating with increased myocardial stiffness (Borbély et al. 2009; Hudson et al. 2011; Hamdani et al. 2013a; Kötter et al. 2013; Hopf et al. 2018). These alterations in titin phosphorylation might originate from the aberrant activity of protein kinases observed in pathological conditions (Bowling et al. 1999; van Heerebeek et al. 2012), or by the expression of phosphatases such as protein phosphatase 5, whose levels are increased in heart failure and diastolic dysfunction (Krysiak et al. 2018). Targeting altered titin phosphorylation has been proposed as a therapeutic strategy in heart failure (Bishu et al. 2011; Furukawa et al. 2024; Vahle et al. 2025). The increase in oxidative stress characteristic of several forms of muscle pathology has also been suggested to alter sarcomere function via changes in oxidative PTMs (Avner et al. 2012). Indeed, increased titin oxidation has been detected in failing (Tomin et al. 2021) and ischemic hearts (Loescher et al. 2020; Töpf et al. 2024) and has been proposed to contribute to altered cardiomyocyte mechanics. A similar scenario has been proposed for skeletal muscle subject to immobilization (Watanabe et al. 2025).

Finally, titin integrity is compromised in several pathological contexts including ischemic disease, anthracycline-induced cardiotoxicity, and atrial fibrillation (Ali et al. 2010; Kötter et al. 2016; Chan et al. 2021; Cizauskas et al. 2024), which are conditions in which a cleaved form of titin (known as T2) has been detected. Interestingly, the inhibition of matrix metalloprotease 2 (MMP-2) prevented both cleavage of titin and myocardial fibrosis in experimental models of anthracycline-induced cardiotoxicity (Chan et al. 2021). However, the extent to which titin cleavage directly contributes to myocardial remodeling remains to be fully addressed, given that MMP-2 also targets other sarcomeric and non-sarcomeric proteins. As discussed in the next section, new precise models that selectively compromise titin integrity are beginning to clarify this issue.

## Models of titin genetic disease: pathophysiology and therapeutic strategies

Despite the limitations of functional assays outlined in the “Current challenges in the clinical interpretation of titin variants” section, animal and in vitro models have provided insights into the physiopathology and potential treatment strategies for conditions induced by *TTN* variants. For instance, in vitro experiments using hiPSCs-derived cardiomyocyte models containing DCM-associated *TTN*tvs variants showed that CRISPR-mediated reading frame repair provides functional recovery (Romano et al. 2022) and that CRISPR-directed activation of the *TTN* promoter rescues sarcomere deficits in hiPSC-derived models of DCM (Ghahremani et al. 2024). Gramlich et al. (2015) also showed that antisense oligonucleotide-mediated exon skipping that avoids a titin frameshift mutation improved sarcomeric ultrastructure and cardiac performance in human and murine models. Zebrafish models facilitated the discovery of novel titin isoforms, including Cronos, that allowed a better understanding of titin-driven cardiomyopathies (Zou et al. 2015; Deo 2016), as subsequently confirmed in human cardiomyocytes (Zaunbrecher et al. 2019). Also, models that delete specific regions of titin have been valuable in elucidating titin’s role in cardiac physiology and mechanosignaling as well as its contribution to myocyte passive stiffness (Weinert et al. 2006; Radke et al. 2007, 2019; H. L. Granzier et al. 2009; Chung et al. 2013; Charton et al. 2016; Biquand et al. 2021; Methawasin et al. 2022). Regarding acquired heart disease, preclinical studies have shown that the inhibition of RBM20 and subsequent generation of longer titin isoforms can be used to ameliorate symptoms in heart failure models (Bull et al. 2016; Hinze et al. 2016; Methawasin et al. 2016; Radke et al. 2021; Celik et al. 2025).

In the context of skeletal muscle titinopathies, murine models of TMD/LGMD R10 titin-related myopathies carrying the FinMaj mutation, which increases titin’s susceptibility to calpain cleavage, (Charton et al. 2015), have shown that reducing calpain-3 expression attenuates dystrophic histological features (Charton et al. 2010). Similarly, mdm mice carrying a spontaneous in-frame deletion of 83 out of the 104 amino acids in the N2A segment of titin show a severe form of muscular dystrophy with early death that correlates with altered protein-protein interactions (Garvey et al. 2002; Witt et al. 2004; Huebsch et al. 2005). Other regional knock-outs also result in reduced muscle performance, muscle atrophy, and myonuclei internalization (Gotthardt et al. 2003; Peng et al. 2005; Buck et al. 2014; H. Granzier et al. 2014; Charton et al. 2016; Brynnel et al. 2018; Radke et al. 2019).

Collectively, the mouse models presented above are not only a tool to develop therapeutic strategies for titinopathies, but they also identify titin as a major regulator of myocyte function. However, these models do not yet resolve whether the observed pathophysiological effects arise from the perturbation of titin mechanical function or from non-mechanical signaling roles. This uncertainty has been recently addressed with emerging experimental models that perturb titin integrity in vitro and in vivo by specifically cleaving the protein upon heterologous expression of Tobacco Etch Protease (TEVp) (Rivas-Pardo et al. 2020; Freundt et al. 2025; López-Unzu et al. 2025; Pricolo et al. 2025; Silva-Rojas et al. 2025). Remarkably, these models can offer new mechanistic insights of the contribution of cleaved titin molecules in episodes of cardiac ischemia (Ali et al. 2010; Kötter et al. 2016), anthracycline-derived cardiotoxicity (Chan et al. 2021), atrial fibrillation (Cizauskas et al. 2024), pulmonary hypertension (López-Unzu et al. 2025), metabolic syndrome (Cuijpers et al. 2021), and, potentially, ACM (Taylor et al. 2011; Anderson et al. 2013). Collectively, studies exploiting TEVp-mediated severing of titin in living myocytes demonstrate that titin cleavage induces sarcomere disassembly and drives both skeletal muscle and cardiac disease. In skeletal muscle, fibers containing cleaved titin remain viable, but undergo atrophy and myonuclei internalization (Silva-Rojas et al. 2025). In cardiac muscle, cleavage of ~ 30% of titin triggers a rapid fibrotic response, accompanied by reduced cell-cell connections between cardiomyocytes (López-Unzu et al. 2025; Pricolo et al. 2025). Importantly, cleaved titin molecules may elicit the same mechanosignaling as truncated molecules observed in genetic diseases since both conditions involve titin recoiling towards the Z-disk (McAfee et al. 2023; Silva-Rojas et al. 2025).

## Perspectives

The field of titin has made a long way since the initial reports on the existence of a very large protein in muscle samples (Maruyama 1976; Wang et al. 1979). Now we know the fundamental functions of the protein and its association with genetic and acquired skeletal muscle and heart disease, but we are still lacking full mechanistic understanding on how the integrative roles of the protein are perturbed by mutations or posttranslational processing. Emerging models of titin dysfunction are expected to improve our understanding of titinopathies and cardiac conditions involving titin; these models should also be useful to test novel therapies before translation to human patients. Unfortunately, murine models of *TTN*tvs have limited value since they show little to no basal phenotype in heterozygosis but are lethal in homozygosis (Gramlich et al. 2009; Schafer et al. 2017). Alternative inducible KO and knock-down models may not recapitulate toxicity of truncated titins; indeed, differently from carriers of *TTN*tvs (Ahlberg et al. 2018; Junttila et al. 2018; Verdonschot et al. 2018), these models applied to cardiac titin result in heart disease without myocardial fibrosis (Liao et al. 2019; Radke et al. 2019). In contrast, murine titin-cleavage models do trigger fibrosis (Freundt et al. 2025; López-Unzu et al. 2025; Silva-Rojas et al. 2025). In this regard, hiPSC-derived cardiomyocytes and their incorporation into engineered tissues together with stromal cells offers an enticing opportunity to study the role of *TTN*tvs in human cells overcoming limitations of mouse models and to provide proof-of-concept of potential therapies. Future research will also need to address why clinical manifestations of pathogenic *TTN* variants vary from no disease to DCM (Herman et al. 2012), primary fibrosis (Junttila et al. 2018) or atrial fibrillation (Ahlberg et al. 2018; X. Jiang et al. 2024). Is this variation dependent on the specific location of the variant? Can the same class of variants cause all these different forms of disease? Do environmental factors play a role in phenotype specification? There also remains an urgent need for the development of methods that can guide pathogenicity assessment of the thousands of titin variants (particularly missense ones and those affecting intronic regions of *TTN* (Kim et al. 2025)) found in patients. Related to this, it is conceivable that mechanisms of pathogenicity of missense titin variants do not fully overlap with those induced by *TTN*tvs, potentially requiring tailored therapeutic interventions. Undoubtedly, mending all possible *Achilles heels* of titin (let them be mutations, sites of proteolysis or aberrant posttranslational modifications) will require the whole warfare of contemporary (personalized) pharmacology (Ware and Cook 2018; Javed and Halliday 2023).

## Data Availability

No datasets were generated or analysed during the current study.

## References

[CR1] Abdellatif M, Trummer-Herbst V, Koser F, Durand S, Adão R, Vasques-Nóvoa F, Freundt JK, Voglhuber J, Pricolo M-R, Kasa M, Türk C, Aprahamian F, Herrero-Galán E, Hofer SJ, Pendl T, Rech L, Kargl J, Anto-Michel N, Ljubojevic-Holzer S, ..., Sedej S (2021) Nicotinamide for the treatment of heart failure with preserved ejection fraction. Sci Transl Med 13(580). 10.1126/scitranslmed.abd7064 10.1126/scitranslmed.abd7064 PMC7611499 33568522

[CR2] Ahlberg G, Refsgaard L, Lundegaard PR, Andreasen L, Ranthe MF, Linscheid N, Nielsen JB, Melbye M, Haunsø S, Sajadieh A, Camp L, Olesen S-P, Rasmussen S, Lundby A, Ellinor PT, Holst AG, Svendsen JH, Olesen MS (2018) Rare truncating variants in the sarcomeric protein titin associate with familial and early-onset atrial fibrillation. Nat Commun 9(1):4316. 10.1038/s41467-018-06618-y 30333491 10.1038/s41467-018-06618-y PMC6193003

[CR3] Ait-Mou Y, Hsu K, Farman GP, Kumar M, Greaser ML, Irving TC, de Tombe PP (2016) Titin strain contributes to the Frank-Starling law of the heart by structural rearrangements of both thin- and thick-filament proteins. Proc Natl Acad Sci U S A 113(8):2306–2311. 10.1073/pnas.1516732113 26858417 10.1073/pnas.1516732113 PMC4776536

[CR4] Akinrinade O, Heliö T, Lekanne Deprez RH, Jongbloed JDH, Boven LG, van den Berg MP, Pinto YM, Alastalo TP, Myllykangas S, Spaendonck-Zwarts K, Tintelen JPvan, van der Zwaag PA, Koskenvuo J (2019) Relevance of titin missense and non-frameshifting insertions/deletions variants in dilated cardiomyopathy. Sci Rep 9(1):1–9. 10.1038/s41598-019-39911-x30626917

[CR5] Alegre-Cebollada J (2021) Protein nanomechanics in biological context. Biophys Rev 13(4):435–454. 10.1007/s12551-021-00822-9 34466164 10.1007/s12551-021-00822-9 PMC8355295

[CR6] Alegre-Cebollada J, Kosuri P, Giganti D, Eckels E, Rivas-Pardo JA, Hamdani N, Warren CM, Solaro RJ, Linke WA, Fernández JM (2014) S-glutathionylation of cryptic cysteines enhances titin elasticity by blocking protein folding. Cell 156(6):1235–1246. 10.1016/j.cell.2014.01.056 24630725 10.1016/j.cell.2014.01.056 PMC3989842

[CR7] Ali MAM, Cho WJ, Hudson B, Kassiri Z, Granzier H, Schulz R (2010) Titin is a target of matrix metalloproteinase-2: implications in myocardial ischemia/reperfusion injury. Circulation 122(20):2039–2047. 10.1161/CIRCULATIONAHA.109.930222 21041693 10.1161/CIRCULATIONAHA.109.930222 PMC3057897

[CR8] Anderson BR, Bogomolovas J, Labeit S, Granzier H (2010) The effects of PKCα phosphorylation on the extensibility of titin’s PEVK element. J Struct Biol. 10.1016/j.jsb.2010.02.002 20149875 10.1016/j.jsb.2010.02.002 PMC2856808

[CR9] Anderson BR, Bogomolovas J, Labeit S, Granzier H (2013) Single molecule force spectroscopy on titin implicates immunoglobulin domain stability as a cardiac disease mechanism. J Biol Chem 288(8):5303–5315. 10.1074/jbc.M112.401372 23297410 10.1074/jbc.M112.401372 PMC3581406

[CR10] Ávila-Polo R, Malfatti E, Lornage X, Cheraud C, Nelson I, Nectoux J, Böhm J, Schneider R, Hedberg-Oldfors C, Eymard B, Monges S, Lubieniecki F, Brochier G, Bui MT, Madelaine A, Labasse C, Beuvin M, Lacène E, Boland A, Thao Bui M, Deleuze JF, Thompson J, Richard I, Taratuto AL, Udd B, Leturcq F, Bonne G, Oldfors A, Laporte J, Romero NB (2018) Loss of sarcomeric scaffolding as a common baseline histopathologic lesion in titin-related myopathies. J Neuropathol Exp Neurol 77(12):1101–1114. 10.1093/JNEN/NLY095 30365001 10.1093/jnen/nly095

[CR11] Avner B, Shioura K, Scruggs S, Grachoff M, Geenen D, Helseth DL Jr, Farjah M, Goldspink P, Solaro RJ (2012) Myocardial infarction in mice alters sarcomeric function via post-translational protein modification. Mol Cell Biochem 363(1):203–215. 10.1007/s11010-011-1172-z 10.1007/s11010-011-1172-z PMC3659404 22160857

[CR12] Balogh-Molnár A, Tordai H, Mártonfalvi Z (2025) Dephosphorylation-induced structural reorganization of skeletal muscle titin molecules. Int J Biol Macromol 322:147085. 10.1016/J.IJBIOMAC.2025.147085 40854385 10.1016/j.ijbiomac.2025.147085

[CR13] Bang M-L, Centner T, Fornoff F, Geach AJ, Gotthardt M, McNabb M, Witt CC, Labeit D, Gregorio CC, Granzier H, Labeit S (2001) The complete gene sequence of titin, expression of an unusual ≈700-kDa titin isoform, and its interaction with obscurin identify a novel Z-line to I-band linking system. Circ Res 89(11):1065–1072. 10.1161/HH2301.100981 11717165 10.1161/hh2301.100981

[CR14] Begay RL, Graw S, Sinagra G, Merlo M, Slavov D, Gowan K, Jones KL, Barbati G, Spezzacatene A, Brun F, Di Lenarda A, Smith JE, Granzier HL, Mestroni L, Taylor M (2015) Role of titin missense variants in dilated cardiomyopathy. J Am Heart Assoc 4(11). 10.1161/JAHA.115.002645 10.1161/JAHA.115.002645 PMC4845231 26567375

[CR15] Bennett P, Rees M, Gautel M (2020) The axial alignment of titin on the muscle thick filament supports its role as a molecular ruler. J Mol Biol 432(17):4815–4829. 10.1016/j.jmb.2020.06.025 32619437 10.1016/j.jmb.2020.06.025 PMC7427331

[CR16] Beqqali A, Bollen IAE, Rasmussen TB, van den Hoogenhof MM, van Deutekom HWM, Schafer S, Haas J, Meder B, Sørensen KE, van Oort RJ, Mogensen J, Hubner N, Creemers EE, van der Velden J, Pinto YM (2016) A mutation in the glutamate-rich region of RNA-binding motif protein 20 causes dilated cardiomyopathy through missplicing of titin and impaired Frank-Starling mechanism. Cardiovasc Res 112(1):452–463. 10.1093/cvr/cvw192 27496873 10.1093/cvr/cvw192

[CR17] Bertz M, Wilmanns M, Rief M (2009) The titin-telethonin complex is a directed, superstable molecular bond in the muscle Z-disk. Proc Natl Acad Sci U S A 106(32):13307–13310. 10.1073/pnas.0902312106 19622741 10.1073/pnas.0902312106 PMC2726412

[CR18] Biquand A, Spinozzi S, Tonino P, Cosette J, Strom J, Elbeck Z, Knöll R, Granzier H, Lostal W, Richard I (2021) Titin M-line insertion sequence 7 is required for proper cardiac function in mice. J Cell Sci. 10.1242/jcs.258684 34401916 10.1242/jcs.258684 PMC8466004

[CR19] Bishu K, Hamdani N, Mohammed SF, Kruger M, Ohtani T, Ogut O, Brozovich FV, Burnett JC, Linke WA, Redfield MM (2011) Sildenafil and B-type natriuretic peptide acutely phosphorylate titin and improve diastolic distensibility in vivo. Circulation 124(25):2882–2891. 10.1161/CIRCULATIONAHA.111.048520 22144574 10.1161/CIRCULATIONAHA.111.048520 PMC3412357

[CR20] Bogomolovas J, Gasch A, Simkovic F, Rigden DJ, Labeit S, Mayans O (2014) Titin kinase is an inactive pseudokinase scaffold that supports MuRF1 recruitment to the sarcomeric M-line. Open Biol 4(5):140041. 10.1098/rsob.140041 24850911 10.1098/rsob.140041 PMC4042850

[CR21] Bogomolovas J, Fleming JR, Anderson BR, Williams R, Lange S, Simon B, Khan MM, Rudolf R, Franke B, Bullard B, Rigden DJ, Granzier H, Labeit S, Mayans O (2016) Exploration of pathomechanisms triggered by a single-nucleotide polymorphism in titin’s I-band: the cardiomyopathy-linked mutation T2580I. Open Biol 6(9):160114. 10.1098/rsob.160114 27683155 10.1098/rsob.160114 PMC5043576

[CR22] Bogomolovas J, Fleming JR, Franke B, Manso B, Simon B, Gasch A, Markovic M, Brunner T, Knöll R, Chen J, Labeit S, Scheffner M, Peter C, Mayans O (2021) Titin kinase ubiquitination aligns autophagy receptors with mechanical signals in the sarcomere. EMBO Rep 22(10):e48018. 10.15252/embr.201948018 34402565 10.15252/embr.201948018 PMC8490993

[CR23] Borbély A, Falcao-Pires I, Van Heerebeek L, Hamdani N, Édes I, Gavina C, Leite-Moreira AF, Bronzwaer JGF, Papp Z, Van Der Velden J, Stienen GJM, Paulus WJ (2009) Hypophosphorylation of the stiff N2B titin isoform raises cardiomyocyte resting tension in failing human myocardium. Circ Res 104(6):780–786. 10.1161/CIRCRESAHA.108.193326 19179657 10.1161/CIRCRESAHA.108.193326

[CR24] Bourfiss M, van Vugt M, Alasiri AI, Ruijsink B, van Setten J, Schmidt AF, Dooijes D, Puyol-Antón E, Velthuis BK, van Tintelen JP, te Riele ASJM, Baas AF, Asselbergs FW (2022) Prevalence and disease expression of pathogenic and likely pathogenic variants associated with inherited cardiomyopathies in the general population. Circ Genom Precis Med 15(6):E003704. 10.1161/CIRCGEN.122.003704 36264615 10.1161/CIRCGEN.122.003704 PMC9770140

[CR25] Bowling N, Walsh RA, Song G, Estridge T, Sandusky GE, Fouts RL, Mintze K, Pickard T, Roden R, Bristow MR, Sabbah HN, Mizrahi JL, Gromo G, King GL, Vlahos CJ (1999) Increased protein kinase C activity and expression of Ca2+-sensitive isoforms in the failing human heart. Circulation 99(3):384–391. 10.1161/01.CIR.99.3.384 9918525 10.1161/01.cir.99.3.384

[CR26] Brauch KM, Karst ML, Herron KJ, de Andra M, Pellikka PA, Rodeheffer RJ, Michels VV, Olson TM (2009) Mutations in ribonucleic acid binding protein gene cause familial dilated cardiomyopathy. J Am Coll Cardiol 54(10):930–941. 10.1016/j.jacc.2009.05.038 19712804 10.1016/j.jacc.2009.05.038 PMC2782634

[CR27] Bryen SJ, Ewans LJ, Pinner J, MacLennan SC, Donkervoort S, Castro D, Töpf A, O’Grady G, Cummings B, Chao KR, Weisburd B, Francioli L, Faiz F, Bournazos AM, Hu Y, Grosmann C, Malicki DM, Doyle H, Witting N, ..., Cooper ST (2020) Recurrent TTN metatranscript-only c.39974-11T>G splice variant associated with autosomal recessive arthrogryposis multiplex congenita and myopathy. Hum Mutat 41(2):403–411. 10.1002/humu.23938 10.1002/humu.23938 PMC7306402 31660661

[CR28] Brynnel A, Hernandez Y, Kiss B, Lindqvist J, Adler M, Kolb J, van der Pijl R, Gohlke J, Strom J, Smith J, Ottenheijm C, Granzier HL (2018) Downsizing the molecular spring of the giant protein titin reveals that skeletal muscle titin determines passive stiffness and drives longitudinal hypertrophy. ELife 7. 10.7554/eLife.40532 10.7554/eLife.40532 PMC6300359 30565562

[CR29] Buck D, Smith JE, Chung CS, Ono Y, Sorimachi H, Labeit S, Granzier HL (2014) Removal of immunoglobulin-like domains from titin’s spring segment alters titin splicing in mouse skeletal muscle and causes myopathy. J Gen Physiol 143(2):215–230. 10.1085/jgp.201311129 24470489 10.1085/jgp.201311129 PMC4001778

[CR30] Bull M, Methawasin M, Strom J, Nair P, Hutchinson K, Granzier H (2016) Alternative splicing of titin restores diastolic function in an HFpEF-like genetic murine model (TtnΔIAjxn). Circ Res 119(6):764–772. 10.1161/CIRCRESAHA.116.308904 27470639 10.1161/CIRCRESAHA.116.308904 PMC5059842

[CR31] Bullard B, Ferguson C, Minajeva A, Leake MC, Gautel M, Labeit D, Ding L, Labeit S, Horwitz J, Leonard KR, Linke WA (2004) Association of the chaperone αβ-crystallin with titin in heart muscle. J Biol Chem 279(9):7917–7924. 10.1074/jbc.M307473200 14676215 10.1074/jbc.M307473200

[CR32] Cazorla O, Freiburg A, Helmes M, Centner T, McNabb M, Wu Y, Trombitás K, Labeit S, Granzier H (2000) Differential expression of cardiac titin isoforms and modulation of cellular stiffness. Circ Res 86(1):59–67. 10.1161/01.RES.86.1.59 10625306 10.1161/01.res.86.1.59

[CR33] Celik S, Hyrefelt L, Czuba T, Li Y, Assis J, Martinez J, Johansson M, André O, Synnergren J, Sandstedt J, Nordenfelt P, Vukusic K, Smith JG, Gidlöf O (2025) Antisense-mediated regulation of exon usage in the elastic spring region of titin modulates sarcomere function. Cardiovasc Res 121(4):629–642. 10.1093/CVR/CVAF037 40042822 10.1093/cvr/cvaf037 PMC12054628

[CR34] Ceyhan-Birsoy O, Agrawal PB, Hidalgo C, Schmitz-Abe K, DeChene ET, Swanson LC, Soemedi R, Vasli N, Iannaccone ST, Shieh PB, Shur N, Dennison JM, Lawlor MW, Laporte J, Markianos K, Fairbrother WG, Granzier H, Beggs AH (2013) Recessive truncating titin gene, TTN, mutations presenting as centronuclear myopathy. Neurology 81(14):1205–1214. 10.1212/WNL.0b013e3182a6ca62 23975875 10.1212/WNL.0b013e3182a6ca62 PMC3795603

[CR35] Chan BYH, Roczkowsky A, Cho WJ, Poirier M, Sergi C, Keschrumrus V, Churko JM, Granzier H, Schulz R (2021) MMP inhibitors attenuate doxorubicin cardiotoxicity by preventing intracellular and extracellular matrix remodelling. Cardiovasc Res 117(1):188–200. 10.1093/CVR/CVAA017 31995179 10.1093/cvr/cvaa017 PMC7797218

[CR36] Charton K, Danièle N, Vihola A, Roudaut C, Gicquel E, Monjaret F, Tarrade A, Sarparanta J, Udd B, Richard I (2010) Removal of the calpain 3 protease reverses the myopathology in a mouse model for titinopathies. Hum Mol Genet 19(23):4608–4624. 10.1093/HMG/DDQ388 20855473 10.1093/hmg/ddq388

[CR37] Charton K, Sarparanta J, Vihola A, Milic A, Jonson PH, Suel L, Luque H, Boumela I, Richard I, Udd B (2015) CAPN3-mediated processing of C-terminal titin replaced by pathological cleavage in titinopathy. Hum Mol Genet 24(13):3718–3731. 10.1093/HMG/DDV116 25877298 10.1093/hmg/ddv116

[CR38] Charton K, Suel L, Henriques SF, Moussu JP, Bovolenta M, Taillepierre M, Becker C, Lipson K, Richard I (2016) Exploiting the CRISPR/Cas9 system to study alternative splicing in vivo: application to titin. Hum Mol Genet 25(20):4518–4532. 10.1093/HMG/DDW280 28173117 10.1093/hmg/ddw280

[CR39] Chauveau C, Bonnemann CG, Julien C, Kho AL, Marks H, Talim B, Maury P, Arne-Bes MC, Uro-Coste E, Alexandrovich A, Vihola A, Schafer S, Kaufmann B, Medne L, Hübner N, Foley AR, Santi M, Udd B, Topaloglu H, Ferreiro A (2014) Recessive TTN truncating mutations define novel forms of core myopathy with heart disease. Hum Mol Genet 23(4):980–991. 10.1093/HMG/DDT494 24105469 10.1093/hmg/ddt494 PMC3954110

[CR40] Chinnery PF, Johnson MA, Walls TJ, John Gibson G, Fawcett PRW, Jamieson S, Fulthorpe JJ, Cullen M, Hudgson P, Bushby KMD (2001) A novel autosomal dominant distal myopathy with early respiratory failure: clinico-pathologic characteristics and exclusion of linkage to candidate genetic loci. Ann Neurol 49(4):443–452. 10.1002/ANA.93 11310621

[CR41] Choi SH, Weng L-C, Roselli C, Lin H, Haggerty CM, Shoemaker MB, Barnard J, Arking DE, Chasman DI, Albert CM, Chaffin M, Tucker NR, Smith JD, Gupta N, Gabriel S, Margolin L, Shea MA, Shaffer CM, Yoneda ZT, Ellinor PT (2018) Association between titin loss-of-function variants and early-onset atrial fibrillation. JAMA 320(22):2354. 10.1001/jama.2018.18179 30535219 10.1001/jama.2018.18179 PMC6436530

[CR42] Chung CS, Hutchinson KR, Methawasin M, Saripalli C, Smith JE, Hidalgo CG, Luo X, Labeit S, Guo C, Granzier HL (2013) Shortening of the elastic tandem immunoglobulin segment of titin leads to diastolic dysfunction. Circulation 128(1):19–28. 10.1161/CIRCULATIONAHA.112.001268 23709671 10.1161/CIRCULATIONAHA.112.001268 PMC3822017

[CR43] Cizauskas HE, Burnham HV, Panni A, Peña A, Alvarez-Arce A, Davis MT, Araujo KN, Delligatti CE, Edassery S, Kirk JA, Arora R, Barefield DY (2024) Proteolytic degradation of atrial sarcomere proteins underlies contractile defects in atrial fibrillation. Am J Physiol Heart Circ Physiol 327(2):H460–H472. 10.1152/AJPHEART.00148.2024 38940916 10.1152/ajpheart.00148.2024 PMC11442024

[CR44] Coppens S, Deconinck N, Sullivan P, Smolnikov A, Clayton JS, Griffin KR, Jones KJ, Vilain CN, Kadhim H, Bryen SJ, Faiz F, Waddell LB, Evesson FJ, Bakshi M, Pinner JR, Charlton A, Brammah S, Graf NS, Krivanek M, Oates EC (2025) Congenital titinopathy: comprehensive characterization of the most severe end of the disease spectrum. Ann Neurol 97(4):611–628. 10.1002/ana.27087 39853809 10.1002/ana.27087 PMC11889535

[CR45] Cuijpers I, Papageorgiou AP, Carai P, Herwig M, Mügge A, Klein T, Hamdani N, Jones EAV, Heymans S (2021) Linagliptin prevents left ventricular stiffening by reducing titin cleavage and hypophosphorylation. J Cell Mol Med 25(2):729–741. 10.1111/JCMM.16122 33295687 10.1111/jcmm.16122 PMC7812306

[CR46] da Silva Lopes K, Pietas A, Radke MH, Gotthardt M (2011) Titin visualization in real time reveals an unexpected level of mobility within and between sarcomeres. J Cell Biol 193(4):785–798. 10.1083/JCB.201010099 21555460 10.1083/jcb.201010099 PMC3166869

[CR47] De Cid R, Ben Yaou R, Roudaut C, Charton K, Baulande S, Leturcq F, Romero NB, Malfatti E, Beuvin M, Vihola A, Criqui A, Nelson I, Nectoux J, Ben Aim L, Caloustian C, Olaso R, Udd B, Bonne G, Eymard B, Richard I (2015) A new titinopathy: childhood-juvenile onset Emery-Dreifuss-like phenotype without cardiomyopathy. Neurology 85(24):2126–2135. 10.1212/WNL.0000000000002200 26581302 10.1212/WNL.0000000000002200 PMC4691685

[CR48] Deo RC (2016) Alternative splicing, internal promoter, nonsense-mediated decay, or all three: explaining the distribution of truncation variants in titin. Circ Cardiovasc Genet 9(5):419–425. 10.1161/CIRCGENETICS.116.001513 27625338 10.1161/CIRCGENETICS.116.001513 PMC5068190

[CR49] Desai D, Sekhon A, Tiessen C, Mebrahtu A, Herzog W (2025) Beyond calcium: cross-bridge binding regulates titin’s contribution to muscle force. J Exp Biol. 10.1242/jeb.250931 10.1242/jeb.250931 41340596

[CR50] Di Feo MF, Oghabian A, Nippala E, Gautel M, Jungbluth H, Forzano F, Malfatti E, Castiglioni C, Krey I, Gomez Andres D, Brady AF, Iascone M, Cereda A, Pezzani L, Natera De Benito D, Nascimiento Osorio A, Estévez Arias B, Kurbatov SA, Attie-Bitach T, ..., Savarese M (2024) Inferring disease course from differential exon usage in the wide titinopathy spectrum. Ann Clin Transl Neurol 11(10):2745–2755. 10.1002/acn3.52189 10.1002/acn3.52189 PMC11514934 39198997

[CR51] Domínguez F, Lalaguna L, Martínez-Martín I, Piqueras-Flores J, Rasmussen TB, Zorio E, Giovinazzo G, Prados B, Ochoa JP, Bornstein B, González-López E, Velázquez-Carreras D, Pricolo MR, Gutiérrez-Agüera F, Bernal JA, Herrero-Galán E, Alegre-Cebollada J, Lara-Pezzi E, García-Pavía P (2023) Titin missense variants as a cause of familial dilated cardiomyopathy. Circulation 147(22):1711–1713. 10.1161/CIRCULATIONAHA.122.062833 37253077 10.1161/CIRCULATIONAHA.122.062833

[CR52] Douvdevany G, Erlich I, Haimovich-Caspi L, Mashiah T, Prondzynski M, Pricolo MR, Alegre-Cebollada J, Linke WA, Carrier L, Kehat I (2024) Imaging of existing and newly translated proteins elucidates mechanisms of sarcomere turnover. Circ Res 135(4):474–487. 10.1161/CIRCRESAHA.123.323819 38962864 10.1161/CIRCRESAHA.123.323819

[CR53] Dowling JJ, Weihl CC, Spencer MJ (2021) Molecular and cellular basis of genetically inherited skeletal muscle disorders. Nat Rev Mol Cell Biol 22(11):713–732. 10.1038/s41580-021-00389-z 34257452 10.1038/s41580-021-00389-z PMC9686310

[CR54] Dutta D, Nguyen V, Campbell KS, Padrón R, Craig R (2023) Cryo-EM structure of the human cardiac myosin filament. Nature 623(7988):853–862. 10.1038/s41586-023-06691-4 37914935 10.1038/s41586-023-06691-4 PMC10846670

[CR55] Eckels EC, Tapia-Rojo R, Rivas-Pardo JA, Fernández JM (2018) The work of titin protein folding as a major driver in muscle contraction. Annu Rev Physiol 80(1):327–351. 10.1146/annurev-physiol-021317-121254 29433413 10.1146/annurev-physiol-021317-121254 PMC5957538

[CR56] Eckels EC, Haldar S, Tapia-Rojo R, Rivas-Pardo JA, Fernández JM (2019) The mechanical power of titin folding. Cell Rep 27(6):1836-1847.e4. 10.1016/j.celrep.2019.04.046 31067467 10.1016/j.celrep.2019.04.046 PMC6937205

[CR57] Fernández-Marmiesse A, Carrascosa-Romero MC, Alfaro Ponce B, Nascimento A, Ortez C, Romero N, Palacios L, Jimenez-Mallebrera C, Jou C, Gouveia S, Couce ML (2017) Homozygous truncating mutation in prenatally expressed skeletal isoform of TTN gene results in arthrogryposis multiplex congenita and myopathy without cardiac involvement. Neuromuscul Disord 27(2):188–192. 10.1016/j.nmd.2016.11.002 28040389 10.1016/j.nmd.2016.11.002

[CR58] Fomin A, Gärtner A, Cyganek L, Tiburcy M, Tuleta I, Wellers L, Folsche L, Hobbach AJ, von Frieling-Salewsky M, Unger A, Hucke A, Koser F, Kassner A, Sielemann K, Streckfuß-Bömeke K, Hasenfuss G, Goedel A, Laugwitz KL, Moretti A, …, Linke WA (2021) Truncated titin proteins and titin haploinsufficiency are targets for functional recovery in human cardiomyopathy due to *TTN* mutations. Sci Transl Med 13(618):1–16. 10.1126/scitranslmed.abd3079 10.1126/scitranslmed.abd3079 34731013

[CR59] Freiburg A, Trombitas K, Hell W, Cazorla O, Fougerousse F, Centner T, Kolmerer B, Witt C, Beckmann JS, Gregorio CC, Granzier H, Labeit S (2000) Series of exon-skipping events in the elastic spring region of titin as the structural basis for myofibrillar elastic diversity. Circ Res 86(11):1114–1121. 10.1161/01.RES.86.11.1114 10850961 10.1161/01.res.86.11.1114

[CR60] Freundt JK, Hartmann P, Loescher CM, Unger A, Koser F, Klotz AJ, Wachsmuth L, Hille S, Door MM, Helfen A, Holtmeier R, Faber C, Kirk JA, Hoerr V, Müller OJ, Linke WA (2025) Titin cleavage impairs cardiac mechanical connectivity to drive diastolic failure and fibrosis. bioRxiv 2025.04.21.649485. 10.1101/2025.04.21.649485

[CR61] Fukuzawa A, Lange S, Holt M, Vihola A, Carmignac V, Ferreiro A, Udd B, Gautel M (2008) Interactions with titin and myomesin target obscurin and obscurin-like 1 to the M-band – implications for hereditary myopathies. J Cell Sci 121(11):1841–1851. 10.1242/JCS.028019 18477606 10.1242/jcs.028019

[CR62] Furukawa N, Matsui H, Sunaga H, Nagata K, Hirayama M, Obinata H, Yokoyama T, Ohno K, Kurabayashi M, Koitabashi N (2024) Sacubitril/valsartan improves diastolic left ventricular stiffness with increased titin phosphorylation via cGMP-PKG activation in diabetic mice. Scientific Reports 14(1):25081. 10.1038/S41598-024-75757-8 39443532 10.1038/s41598-024-75757-8 PMC11499646

[CR63] Garcia-Pavia P, Kim Y, Restrepo-Cordoba MA, Lunde IG, Wakimoto H, Smith AM, Toepfer CN, Getz K, Gorham J, Patel P, Ito K, Willcox JA, Arany Z, Li J, Owens AT, Govind R, Nuñez B, Mazaika E, Bayes-Genis A, Walsh R, Finkelman B, Lupon J, Whiffin N, Serrano I, Midwinter W, Wilk A, Bardaji A, Ingold N, Buchan R, Tayal U, Pascual-Figal DA, de Marvao A, Ahmad M, Garcia-Pinilla JM, Pantazis A, Dominguez F, John Baksi A, O’Regan DP, Rosen SD, Prasad SK, Lara-Pezzi E, Provencio M, Lyon AR, Alonso-Pulpon L, Cook SA, DePalma SR, Barton PJR, Aplenc R, Seidman JG, Ky B, Ware JS, Seidman CE (2019) Genetic variants associated with cancer therapy-induced cardiomyopathy. Circulation 140(1):31–41. 10.1161/CIRCULATIONAHA.118.037934 30987448 10.1161/CIRCULATIONAHA.118.037934 PMC6613726

[CR64] Garvey SM, Rajan C, Lerner AP, Frankel WN, Cox GA (2002) The muscular dystrophy with myositis (mdm) mouse mutation disrupts a skeletal muscle-specific domain of titin. Genomics 79(2):146–149. 10.1006/geno.2002.6685 11829483 10.1006/geno.2002.6685

[CR65] Gautel M, Djinović-Carugo K (2016) The sarcomeric cytoskeleton: from molecules to motion. J Exp Biol 219(2):135–145. 10.1242/jeb.124941 26792323 10.1242/jeb.124941

[CR66] Gerull B, Gramlich M, Atherton J, McNabb M, Trombitás K, Sasse-Klaassen S, Seidman JG, Seidman C, Granzier H, Labeit S, Frenneaux M, Thierfelder L (2002) Mutations of *TTN*, encoding the giant muscle filament titin, cause familial dilated cardiomyopathy. Nat Genet 30(2):201–204. 10.1038/ng815 11788824 10.1038/ng815

[CR67] Ghahremani S, Kanwal A, Pettinato A, Ladha F, Legere N, Thakar K, Zhu Y, Tjong H, Wilderman A, Stump WT, Greenberg L, Greenberg MJ, Cotney J, Wei C-L, Hinson JT (2024) CRISPR activation reverses haploinsufficiency and functional deficits caused by TTN truncation variants. Circulation 149(16):1285–1297. 10.1161/CIRCULATIONAHA.123.063972 38235591 10.1161/CIRCULATIONAHA.123.063972 PMC11031707

[CR68] Giganti D, Yan K, Badilla CL, Fernandez JM, Alegre-Cebollada J (2018) Disulfide isomerization reactions in titin immunoglobulin domains enable a mode of protein elasticity. Nat Commun 9(1):185. 10.1038/s41467-017-02528-7 29330363 10.1038/s41467-017-02528-7 PMC5766482

[CR69] Golenhofen N, Arbeiter A, Koob R, Drenckhahn D (2002) Ischemia-induced association of the stress protein αB-crystallin with I-band portion of cardiac titin. J Mol Cell Cardiol 34(3):309–319. 10.1006/jmcc.2001.1513 11945023 10.1006/jmcc.2001.1513

[CR70] Gotthardt M, Hammer RE, Hübner N, Monti J, Witt CC, McNabb M, Richardson JA, Granzier H, Labeit S, Herz J (2003) Conditional expression of mutant M-line titins results in cardiomyopathy with altered sarcomere structure. J Biol Chem 278(8):6059–6065. 10.1074/jbc.M211723200 12464612 10.1074/jbc.M211723200

[CR71] Gramlich M, Michely B, Krohne C, Heuser A, Erdmann B, Klaassen S, Hudson B, Magarin M, Kirchner F, Todiras M, Granzier H, Labeit S, Thierfelder L, Gerull B (2009) Stress-induced dilated cardiomyopathy in a knock-in mouse model mimicking human titin-based disease. J Mol Cell Cardiol 47(3):352–358. 10.1016/J.YJMCC.2009.04.014 19406126 10.1016/j.yjmcc.2009.04.014 PMC2764556

[CR72] Gramlich M, Pane LS, Zhou Q, Chen Z, Murgia M, Schötterl S, Goedel A, Metzger K, Brade T, Parrotta E, Schaller M, Gerull B, Thierfelder L, Aartsma‐Rus A, Labeit S, Atherton JJ, McGaughran J, Harvey RP, Sinnecker D, ..., Moretti A (2015) Antisense-mediated exon skipping: a therapeutic strategy for titin-based dilated cardiomyopathy. EMBO Mol Med 7(5):562–576. 10.15252/EMMM.201505047 10.15252/emmm.201505047 PMC4492817 25759365

[CR73] Granzier HL, Labeit S (2025) Discovery of titin and its role in heart function and disease. Circ Res 136(1):135–157. 10.1161/CIRCRESAHA.124.323051 39745989 10.1161/CIRCRESAHA.124.323051

[CR74] Granzier HL, Radke MH, Peng J, Westermann D, Nelson OL, Rost K, King NMP, Yu Q, Tschöpe C, McNabb M, Larson DF, Labeit S, Gotthardt M (2009) Truncation of titin’s elastic PEVK region leads to cardiomyopathy with diastolic dysfunction. Circ Res 105(6):557–564. 10.1161/CIRCRESAHA.109.200964 19679835 10.1161/CIRCRESAHA.109.200964 PMC2785004

[CR75] Granzier H, Hutchinson KR, Tonino P, Methawasin M, Li FW, Slater RE, Bull MM, Saripalli C, Pappas CT, Gregorio CC, Smith JE, Seidman CE (2014) Deleting titin’s I-band/A-band junction reveals critical roles for titin in biomechanical sensing and cardiac function. Proc Natl Acad Sci U S A 111(40):14589–14594. 10.1073/pnas.1411493111 25246556 10.1073/pnas.1411493111 PMC4210014

[CR76] Grimes KM, Prasad V, McNamara JW (2019) Supporting the heart: functions of the cardiomyocyte’s non-sarcomeric cytoskeleton. J Mol Cell Cardiol 131:187–196. 10.1016/j.yjmcc.2019.04.002 30978342 10.1016/j.yjmcc.2019.04.002 PMC6581584

[CR77] Grison M, Merkel U, Kostan J, Djinović-Carugo K, Rief M (2017) α-actinin/titin interaction: a dynamic and mechanically stable cluster of bonds in the muscle Z-disk. Proc Natl Acad Sci 114(5):1015–1020. 10.1073/pnas.1612681114 28096424 10.1073/pnas.1612681114 PMC5293040

[CR78] Grützner A, Garcia-Manyes S, Kötter S, Badilla CL, Fernandez JM, Linke WA (2009) Modulation of titin-based stiffness by disulfide bonding in the cardiac titin N2-B unique sequence. Biophys J 97(3):825–834. 10.1016/j.bpj.2009.05.037 19651040 10.1016/j.bpj.2009.05.037 PMC2718153

[CR79] Guo W, Schafer S, Greaser ML, Radke MH, Liss M, Govindarajan T, Maatz H, Schulz H, Li S, Parrish AM, Dauksaite V, Vakeel P, Klaassen S, Gerull B, Thierfelder L, Regitz-Zagrosek V, Hacker TA, Saupe KW, Dec GW, Ellinor PT, MacRae CA, Spallek B, Fischer R, Perrot A, Özcelik C, Saar K, Hubner N, Gotthardt M (2012) RBM20, a gene for hereditary cardiomyopathy, regulates titin splicing. Nat Med 18(5):766–773. 10.1038/nm.2693 22466703 10.1038/nm.2693 PMC3569865

[CR80] Hackman P, Vihola A, Haravuori H, Marchand S, Sarparanta J, de Seze J, Labeit S, Witt C, Peltonen L, Richard I, Udd B (2002) Tibial muscular dystrophy is a titinopathy caused by mutations in *TTN*, the gene encoding the giant skeletal-muscle protein titin. Am J Hum Genet 71(3):492–500. 10.1086/342380 12145747 10.1086/342380 PMC379188

[CR81] Haggerty CM, Damrauer SM, Levin MG, Birtwell D, Carey DJ, Golden AM, Hartzel DN, Hu Y, Judy R, Kelly MA, Kember RL, Lester Kirchner H, Leader JB, Liang L, McDermott-Roe C, Babu A, Morley M, Nealy Z, Person TN, Arany Z (2019) Genomics-first evaluation of heart disease associated with titin-truncating variants. Circulation 140(1):42–54. 10.1161/CIRCULATIONAHA.119.039573 31216868 10.1161/CIRCULATIONAHA.119.039573 PMC6602806

[CR82] Hamdani N, Bishu KG, von Frieling-Salewsky M, Redfield MM, Linke WA (2013a) Deranged myofilament phosphorylation and function in experimental heart failure with preserved ejection fraction. Cardiovasc Res 97(3):464–471. 10.1093/cvr/cvs353 23213108 10.1093/cvr/cvs353

[CR83] Hamdani N, Krysiak J, Kreusser MM, Neef S, Dos Remedios CG, Maier LS, Krüger M, Backs J, Linke WA (2013b) Crucial role for Ca2+/calmodulin-dependent protein kinase-II in regulating diastolic stress of normal and failing hearts via titin phosphorylation. Circ Res 112(4):664–674. 10.1161/CIRCRESAHA.111.300105/-/DC1 23283722 10.1161/CIRCRESAHA.111.300105

[CR84] Hamdani N, Herwig M, Linke WA (2017) Tampering with springs: phosphorylation of titin affecting the mechanical function of cardiomyocytes. In: Biophysical reviews (Vol 9, Issue 3). Springer Verlag, pp 225–237. 10.1007/s12551-017-0263-9 10.1007/s12551-017-0263-9 PMC5498327 28510118

[CR85] Hanashima A, Kimoto M, Ujihara Y, Hashimoto K, Usui Y, Ohira M, Hoshino M, Uesugi K, Witt S, Labeit D, Kimura S, Murayama T, Sakurai T, Labeit S, Mohri S (2025) CCDC141 is a connectin/titin and nesprin-1 binding protein that adapts cardiomyocytes to mechanical stress. Commun Biol 8(1):1693. 10.1038/s42003-025-09093-6 41298937 10.1038/s42003-025-09093-6 PMC12657953

[CR86] Hastings R, de Villiers CP, Hooper C, Ormondroyd L, Pagnamenta A, Lise S, Salatino S, Knight SJL, Taylor JC, Thomson KL, Arnold L, Chatziefthimiou SD, Konarev PV, Wilmanns M, Ehler E, Ghisleni A, Gautel M, Blair E, Watkins H, Gehmlich K (2016) Combination of whole genome sequencing, linkage, and functional studies implicates a missense mutation in titin as a cause of autosomal dominant cardiomyopathy with features of left ventricular noncompaction. Circ Cardiovasc Genet 9(5):426–435. 10.1161/CIRCGENETICS.116.001431 27625337 10.1161/CIRCGENETICS.116.001431 PMC5068189

[CR87] Hedberg C, Toledo AG, Gustafsson CM, Larson G, Oldfors A, Macao B (2014) Hereditary myopathy with early respiratory failure is associated with misfolding of the titin fibronectin III 119 subdomain. Neuromuscul Disord 24(5):373–379. 10.1016/j.nmd.2014.02.003 24636144 10.1016/j.nmd.2014.02.003

[CR88] Herman DS, Lam L, Taylor MRG, Wang L, Teekakirikul P, Christodoulou D, Conner L, DePalma SR, McDonough B, Sparks E, Teodorescu DL, Cirino AL, Banner NR, Pennell DJ, Graw S, Merlo M, Di Lenarda A, Sinagra G, Bos JM, Seidman CE (2012) Truncations of titin causing dilated cardiomyopathy. N Engl J Med 366(7):619–628. 10.1056/nejmoa1110186 22335739 10.1056/NEJMoa1110186 PMC3660031

[CR89] Herrero-Galán E, Martínez-Martín I, Alegre-Cebollada J (2019) Redox regulation of protein nanomechanics in health and disease: lessons from titin. Redox Biol 21:101074. 10.1016/j.redox.2018.101074 30584979 10.1016/j.redox.2018.101074 PMC6305763

[CR90] Herrero-Galán E, Martínez-Martín I, Sánchez-González C, Vicente N, Bonzón-Kulichenko E, Calvo E, Suay-Corredera C, Pricolo MR, Fernández-Trasancos Á, Velázquez-Carreras D, Careaga CB, Abdellatif M, Sedej S, Rainer PP, Giganti D, Pérez-Jiménez R, Vázquez J, Alegre-Cebollada J (2022) Basal oxidation of conserved cysteines modulates cardiac titin stiffness and dynamics. Redox Biol 52:102306. 10.1016/j.redox.2022.102306 35367810 10.1016/j.redox.2022.102306 PMC8971355

[CR91] Hessel AL, Kuehn MN, Palmer BM, Nissen D, Mishra D, Joumaa V, Freundt JK, Ma W, Nishikawa KC, Irving TC, Linke WA (2024) The distinctive mechanical and structural signatures of residual force enhancement in myofibers. Proc Natl Acad Sci U S A 121(52):e2413883121. 10.1073/pnas.2413883121 39680764 10.1073/pnas.2413883121 PMC11670058

[CR92] Hidalgo C, Hudson B, Bogomolovas J, Zhu Y, Anderson B, Greaser M, Labeit S, Granzier H (2009) PKC phosphorylation of titin’s PEVK element. Circ Res 105(7):631–638. 10.1161/CIRCRESAHA.109.198465 19679839 10.1161/CIRCRESAHA.109.198465 PMC2764991

[CR93] Hinson JT, Chopra A, Nafissi N, Polacheck WJ, Benson CC, Swist S, Gorham J, Yang L, Schafer S, Sheng CC, Haghighi A, Homsy J, Hubner N, Church G, Cook SA, Linke WA, Chen CS, Seidman JG, Seidman CE (2015) Titin mutations in iPS cells define sarcomere insufficiency as a cause of dilated cardiomyopathy. Science 349(6251):982–986. 10.1126/science.aaa5458 26315439 10.1126/science.aaa5458 PMC4618316

[CR94] Hinze F, Dieterich C, Radke MH, Granzier H, Gotthardt M (2016) Reducing RBM20 activity improves diastolic dysfunction and cardiac atrophy. J Mol Med (Berl) 94(12):1349–1358. 10.1007/s00109-016-1483-3 27889803 10.1007/s00109-016-1483-3 PMC5143357

[CR95] Hopf A-E, Andresen C, Kötter S, Isić M, Ulrich K, Sahin S, Bongardt S, Röll W, Drove F, Scheerer N, Vandekerckhove L, De Keulenaer GW, Hamdani N, Linke WA, Krüger M (2018) Diabetes-induced cardiomyocyte passive stiffening is caused by impaired insulin-dependent titin modification and can be modulated by neuregulin-1. Circ Res 123(3):342–355. 10.1161/CIRCRESAHA.117.312166 29760016 10.1161/CIRCRESAHA.117.312166

[CR96] Hudson B, Hidalgo C, Saripalli C, Granzier H (2011) Hyperphosphorylation of mouse cardiac titin contributes to transverse aortic constriction-induced diastolic dysfunction. Circ Res 109(8):858–866. 10.1161/CIRCRESAHA.111.246819 21835910 10.1161/CIRCRESAHA.111.246819 PMC3191198

[CR97] Huebsch KA, Kudryashova E, Wooley CM, Sher RB, Seburn KL, Spencer MJ, Cox GA (2005) Mdm muscular dystrophy: interactions with calpain 3 and a novel functional role for titin’s N2A domain. Hum Mol Genet 14(19):2801–2811. 10.1093/hmg/ddi313 16115818 10.1093/hmg/ddi313 PMC1350399

[CR98] Hutchinson KR, Saripalli C, Chung CS, Granzier H (2015) Increased myocardial stiffness due to cardiac titin isoform switching in a mouse model of volume overload limits eccentric remodeling. J Mol Cell Cardiol 79:104–114. 10.1016/j.yjmcc.2014.10.020 25450617 10.1016/j.yjmcc.2014.10.020 PMC4302034

[CR99] Ishikawa T, Kimoto H, Seki A, Shirai M, Uto K, Makiyama T, Kitai T, Mishima H, Trujillano D, Simonet F, Baron E, Lindenbaum P, Kyndt F, Goudal A, Fukushima N, Fujita T, Hatakeyama K, Hagiwara N, Yoshiura K-I, Makita N (2025) Phenotypic spectrum of cardiac conduction disturbance and cardiomyopathy linked to titin canonical splice-site variants. Cardiovasc Res 121(11):1712–1721. 10.1093/cvr/cvaf135 40757694 10.1093/cvr/cvaf135

[CR100] Itoh-Satoh M, Hayashi T, Nishi H, Koga Y, Arimura T, Koyanagi T, Takahashi M, Hohda S, Ueda K, Nouchi T, Hiroe M, Marumo F, Imaizumi T, Yasunami M, Kimura A (2002) Titin mutations as the molecular basis for dilated cardiomyopathy. Biochem Biophys Res Commun 291(2):385–393. 10.1006/bbrc.2002.6448 11846417 10.1006/bbrc.2002.6448

[CR101] Japp AG, Gulati A, Cook SA, Cowie MR, Prasad SK (2016) The diagnosis and evaluation of dilated cardiomyopathy. J Am Coll Cardiol 67(25):2996–3010. 10.1016/j.jacc.2016.03.590 27339497 10.1016/j.jacc.2016.03.590

[CR102] Javed S, Halliday BP (2023) Precision therapy in dilated cardiomyopathy: pipedream or paradigm shift? Cambridge Prisms: Precision Medicine 1:e34. 10.1017/pcm.2023.24 38550947 10.1017/pcm.2023.24 PMC10953759

[CR103] Jiang H, Hooper C, Kelly M, Steeples V, Simon JN, Beglov J, Azad AJ, Leinhos L, Bennett P, Ehler E, Kalisch-Smith JI, Sparrow DB, Fischer R, Heilig R, Isackson H, Ehsan M, Patone G, Huebner N, Davies B, Gehmlich K (2021) Functional analysis of a gene-edited mouse model to gain insights into the disease mechanisms of a titin missense variant. Basic Res Cardiol 116(1):14. 10.1007/s00395-021-00853-z 33637999 10.1007/s00395-021-00853-z PMC7910237

[CR104] Jiang X, Ly OT, Chen H, Zhang Z, Ibarra BA, Pavel MA, Brown GE, Sridhar A, Tofovic D, Swick A, Marszalek R, Vanoye CG, Navales F, George AL, Khetani SR, Rehman J, Gao Y, Darbar D, Saxena A (2024) Transient titin-dependent ventricular defects during development lead to adult atrial arrhythmia and impaired contractility. Iscience 27(7):110395. 10.1016/j.isci.2024.110395 39100923 10.1016/j.isci.2024.110395 PMC11296057

[CR105] Junttila MJ, Holmström L, Pylkäs K, Mantere T, Kaikkonen K, Porvari K, Kortelainen ML, Pakanen L, Kerkelä R, Myerburg RJ, Huikuri HV (2018) Primary myocardial fibrosis as an alternative phenotype pathway of inherited cardiac structural disorders. Circulation 137(25):2716–2726. 10.1161/CIRCULATIONAHA.117.032175 29915098 10.1161/CIRCULATIONAHA.117.032175

[CR106] Kellermayer M, Smith SB, Granzier HL, Bustamante C (1997) Folding-unfolding transitions in single titin molecules characterized with laser tweezers. Science 276(5315):1112–1116. 10.1126/science.276.5315.1112 9148805 10.1126/science.276.5315.1112

[CR107] Kellermayer D, Smith JE, Granzier H (2017) Novex-3, the tiny titin of muscle. Biophys Rev 9(3):201–206. 10.1007/S12551-017-0261-Y 28510117 10.1007/s12551-017-0261-y PMC5498326

[CR108] Kellermayer D, Tordai H, Kiss B, Török G, Péter DM, Sayour AA, Pólos M, Hartyánszky I, Szilveszter B, Labeit S, Gángó A, Bedics G, Bödör C, Radovits T, Merkely B, Kellermayer MSZ (2024) Truncated titin is structurally integrated into the human dilated cardiomyopathic sarcomere. J Clin Invest. 10.1172/JCI169753 37962957 10.1172/JCI169753 PMC10763722

[CR109] Kellermayer D, Șulea CM, Tordai H, Benke K, Pólos M, Ágg B, Stengl R, Csonka M, Radovits T, Merkely B, Szabolcs Z, Kellermayer M, Kiss B (2025) Marfan syndrome cardiomyocytes show excess of titin isoform N2BA and extended sarcomeric M-band. J Gen Physiol. 10.1085/jgp.202413690 40062891 10.1085/jgp.202413690 PMC11893164

[CR110] Kim Y, Kim SW, Saul D, Neyazi M, Schmid M, Wakimoto H, Slaven N, Lee JH, Layton O, Wasson LK, Letendre JH, Xiao F, Ewoldt JK, Gkatzis K, Sommer P, Gobert B, Wiest-Daesslé N, McAfee Q, Singhal N, Lun M, Gorham JM, Arany Z, Sharma A, Toepfer CN, Oudit GY, Pu WT, Dickel DE, Pennacchio LA, Visel A, Chen CS, Seidman JG, Seidman CE (2024) Regulation of sarcomere formation and function in the healthy heart requires a titin intronic enhancer. J Clin Invest. 10.1172/JCI183353 39688912 10.1172/JCI183353 PMC11827849

[CR111] Kim Y, Kim SW, Saul D, Neyazi M, Schmid M, Wakimoto H, Slaven N, Lee JH, Layton O, Wasson LK, Letendre JH, Xiao F, Ewoldt JK, Gkatzis K, Sommer P, Gobert B, Wiest-Daesslé N, McAfee Q, Singhal N, …, Seidman CE (2025) Regulation of sarcomere formation and function in the healthy heart requires a titin intronic enhancer. J Clin Invest 135(4). 10.1172/JCI183353 10.1172/JCI183353 PMC11827849 39688912

[CR112] Knöll R, Hoshijima M, Hoffman HM, Person V, Lorenzen-Schmidt I, Bang M-L, Hayashi T, Shiga N, Yasukawa H, Schaper W, McKenna W, Yokoyama M, Schork NJ, Omens JH, McCulloch AD, Kimura A, Gregorio CC, Poller W, Schaper J, Chien KR (2002) The cardiac mechanical stretch sensor machinery involves a Z disc complex that is defective in a subset of human dilated cardiomyopathy. Cell 111(7):943–955. 10.1016/s0092-8674(02)01226-6 12507422 10.1016/s0092-8674(02)01226-6

[CR113] Koser F, Loescher C, Linke WA (2019) Posttranslational modifications of titin from cardiac muscle: how, where and what for? FEBS J. 10.1111/febs.14854 30989819 10.1111/febs.14854 PMC6850032

[CR114] Koser F, Hobbach AJ, Abdellatif M, Herbst V, Türk C, Reinecke H, Krüger M, Sedej S, Linke WA (2022) Acetylation and phosphorylation changes to cardiac proteins in experimental HFpEF due to metabolic risk reveal targets for treatment. Life Sci 309:120998. 10.1016/j.lfs.2022.120998 36179815 10.1016/j.lfs.2022.120998

[CR115] Kötter S, Gout L, Von Frieling-Salewsky M, Müller AE, Helling S, Marcus K, Dos Remedios C, Linke WA, Krüger M (2013) Differential changes in titin domain phosphorylation increase myofilament stiffness in failing human hearts. Cardiovasc Res 99(4):648–656. 10.1093/cvr/cvt144 23764881 10.1093/cvr/cvt144

[CR116] Kötter S, Unger A, Hamdani N, Lang P, Vorgerd M, Nagel-Steger L, Linke WA (2014) Human myocytes are protected from titin aggregation-induced stiffening by small heat shock proteins. J Cell Biol 204(2):187–202. 10.1083/JCB.201306077 24421331 10.1083/jcb.201306077 PMC3897184

[CR117] Kötter S, Kazmierowska M, Andresen C, Bottermann K, Grandoch M, Gorressen S, Heinen A, Moll JM, Scheller J, Gödecke A, Fischer JW, Schmitt JP, Krüger M (2016) Titin-based cardiac myocyte stiffening contributes to early adaptive ventricular remodeling after myocardial infarction. Circ Res 119(9):1017–1029. 10.1161/CIRCRESAHA.116.309685 27650557 10.1161/CIRCRESAHA.116.309685

[CR118] Kramer PA, Duan J, Gaffrey MJ, Shukla AK, Wang L, Bammler TK, Qian W-J, Marcinek DJ (2018) Fatiguing contractions increase protein S-glutathionylation occupancy in mouse skeletal muscle. Redox Biol 17(C):367–376. 10.1016/j.redox.2018.05.011 29857311 10.1016/j.redox.2018.05.011 PMC6007084

[CR119] Krüger M, Kötter S, Grützner A, Lang P, Andresen C, Redfield MM, Butt E, dos Remedios CG, Linke WA (2009) Protein kinase G modulates human myocardial passive stiffness by phosphorylation of the titin springs. Circ Res 104(1):87–94. 10.1161/CIRCRESAHA.108.184408 19023132 10.1161/CIRCRESAHA.108.184408

[CR120] Krysiak J, Unger A, Beckendorf L, Hamdani N, Von Frieling-Salewsky M, Redfield MM, Dos Remedios CG, Sheikh F, Gergs U, Boknik P, Linke WA (2018) Protein phosphatase 5 regulates titin phosphorylation and function at a sarcomere-associated mechanosensor complex in cardiomyocytes. Nat Commun 9(1):1–14. 10.1038/s41467-017-02483-3 29343782 10.1038/s41467-017-02483-3 PMC5772059

[CR121] Labeit S, Gautel M, Lakey A, Trinick J (1992) Towards a molecular understanding of titin. EMBO J 11(5):1711–1716. 10.1002/j.1460-2075.1992.tb05222.x 1582406 10.1002/j.1460-2075.1992.tb05222.x PMC556628

[CR122] Labeit D, Watanabe K, Witt C, Fujita H, Wu Y, Lahmers S, Funck T, Labeit S, Granzier H (2003) Calcium-dependent molecular spring elements in the giant protein titin. Proc Natl Acad Sci U S A 100(23):13716–13721. 10.1073/pnas.2235652100 14593205 10.1073/pnas.2235652100 PMC263879

[CR123] Labeit S, Lahmers S, Burkart C, Fong C, McNabb M, Witt S, Witt C, Labeit D, Granzier H (2006) Expression of distinct classes of titin isoforms in striated and smooth muscles by alternative splicing, and their conserved interaction with filamins. J Mol Biol 362(4):664–681. 10.1016/J.JMB.2006.07.077 16949617 10.1016/j.jmb.2006.07.077

[CR124] Laddach A, Gautel M, Fraternali F (2017) TITINdb-a computational tool to assess titin’s role as a disease gene. Bioinformatics (Oxford, England) 33(21):3482–3485. 10.1093/bioinformatics/btx424 29077808 10.1093/bioinformatics/btx424 PMC5860166

[CR125] Lahmers S, Wu Y, Call DR, Labeit S, Granzier H (2004) Developmental control of titin isoform expression and passive stiffness in fetal and neonatal myocardium. Circ Res 94(4):505–513. 10.1161/01.RES.0000115522.52554.86 14707027 10.1161/01.RES.0000115522.52554.86

[CR126] Lange S, Xiang F, Yakovenko A, Vihola A, Hackman P, Rostkova E, Kristensen J, Brandmeier B, Franzen G, Hedberg B, Gunnarsson LG, Hughes SM, Marchand S, Sejersen T, Richard I, Edström L, Ehler E, Udd B, Gautel M (2005) The kinase domain of titin controls muscle gene expression and protein turnover. Science (New York, Ny) 308(5728):1599–1603. 10.1126/science.1110463 10.1126/science.1110463 15802564

[CR127] Lanzicher T, Zhou T, Saripalli C, Keschrumrus V, Smith JE Iii, Mayans O, Sbaizero O, Granzier H (2020) Single-molecule force spectroscopy on the N2A element of titin: effects of phosphorylation and CARP. Front Physiol 11:173. 10.3389/fphys.2020.00173 10.3389/fphys.2020.00173 PMC7093598 32256378

[CR128] Leite FS, Minozzo FC, Kalganov A, Cornachione AS, Cheng Y-S, Leu NA, Han X, Saripalli C, Yates JR, Granzier H, Kashina AS, Rassier DE (2016) Reduced passive force in skeletal muscles lacking protein arginylation. Am J Physiol Cell Physiol 310(2):C127–C135. 10.1152/ajpcell.00269.2015 26511365 10.1152/ajpcell.00269.2015 PMC4719035

[CR129] LeWinter MM, Granzier HL (2014) Cardiac titin and heart disease. J Cardiovasc Pharmacol 63(3):207–212. 10.1097/FJC.0000000000000007 24072177 10.1097/FJC.0000000000000007 PMC4268868

[CR130] Li H, Linke WA, Oberhauser AF, Carrion-Vazquez M, Kerkvliet JG, Lu H, Marszalek PE, Fernandez JM (2002) Reverse engineering of the giant muscle protein titin. Nature 418(6901):998–1002. 10.1038/nature00938 12198551 10.1038/nature00938

[CR131] Li D, Morales A, Gonzalez-Quintana J, Norton N, Siegfried JD, Hofmeyer M, Hershberger RE (2010) Identification of novel mutations in RBM20 in patients with dilated cardiomyopathy. Clin Transl Sci 3(3):90–97. 10.1111/J.1752-8062.2010.00198.X 20590677 10.1111/j.1752-8062.2010.00198.x PMC2898174

[CR132] Li S, Guo W, Dewey CN, Greaser ML (2013) Rbm20 regulates titin alternative splicing as a splicing repressor. Nucleic Acids Res 41(4):2659–2672. 10.1093/nar/gks1362 23307558 10.1093/nar/gks1362 PMC3575840

[CR133] Li Y, Hessel AL, Unger A, Ing D, Recker J, Koser F, Freundt JK, Linke WA (2020) Graded titin cleavage progressively reduces tension and uncovers the source of A-band stability in contracting muscle. ELife 9. 10.7554/elife.64107 10.7554/eLife.64107 PMC7781594 33357376

[CR134] Li M, Li F, Cheng Z, Cheng J, Wu Q, Wang Z, Wang F, Zhou P (2024) Biallelic truncating TTN variants in M-band encoding exons cause a fetal lethal titinopathy. Prenat Diagn 44(1):81–87. 10.1002/PD.6491 38148006 10.1002/pd.6491

[CR135] Liao D, Chen W, Tan CY, Wong JX, Chan PS, Tan LW, Foo R, Jiang J (2019) Upregulation of Yy1 suppresses dilated cardiomyopathy caused by Ttn insufficiency. Sci Rep 9(1):1–12. 10.1038/s41598-019-52796-0 31705051 10.1038/s41598-019-52796-0 PMC6841687

[CR136] Linke WA (2023) Stretching the story of titin and muscle function. J Biomech 152:111553. 10.1016/j.jbiomech.2023.111553 36989971 10.1016/j.jbiomech.2023.111553

[CR137] Linke WA, Hamdani N (2014) Gigantic business. Circ Res 114(6):1052–1068. 10.1161/CIRCRESAHA.114.301286 24625729 10.1161/CIRCRESAHA.114.301286

[CR138] Linke WA, Stockmeier MR, Ivemeyer M, Hosser H, Mundel P (1998) Characterizing titin’s I-band Ig domain region as an entropic spring. J Cell Sci 111(11):1567–1574. 10.1242/JCS.111.11.1567 9580564 10.1242/jcs.111.11.1567

[CR139] Linke WA, Kulke M, Li H, Fujita-Becker S, Neagoe C, Manstein DJ, Gautel M, Fernandez JM (2002) PEVK domain of titin: an entropic spring with actin-binding properties. J Struct Biol 137(1–2):194–205. 10.1006/jsbi.2002.4468 12064946 10.1006/jsbi.2002.4468

[CR140] Loescher CM, Breitkreuz M, Li Y, Nickel A, Unger A, Dietl A, Schmidt A, Mohamed BA, Kötter S, Schmitt JP, Krüger M, Krüger M, Toischer K, Maack C, Leichert LI, Hamdani N, Linke WA (2020) Regulation of titin-based cardiac stiffness by unfolded domain oxidation (UnDOx). Proc Natl Acad Sci USA 117(39):24545–24556. 10.1073/pnas.2004900117 32929035 10.1073/pnas.2004900117 PMC7533878

[CR141] Loescher CM, Hobbach AJ, Linke WA (2022) Titin (*TTN*): from molecule to modifications, mechanics, and medical significance. Cardiovasc Res 118(14):2903–2918. 10.1093/cvr/cvab328 34662387 10.1093/cvr/cvab328 PMC9648829

[CR142] Loescher CM, Freundt JK, Unger A, Hessel AL, Kühn M, Koser F, Linke WA (2023) Titin governs myocardial passive stiffness with major support from microtubules and actin and the extracellular matrix. Nat Cardiovasc Res 2(11):991–1002. 10.1038/s44161-023-00348-1 39196092 10.1038/s44161-023-00348-1 PMC11358001

[CR143] López-Unzu MA, Pricolo MR, Silva-Rojas R, Clemente-Manteca A, Sánchez-Ortiz D, Vicente N, Velázquez-Carreras D, Morales C, Gavilán-Herrera M, Sen-Martín L, Fernández-Trasancos Á, Labrador-Cantarero V, Sánchez-García L, Relaño-Rupérez C, Sánchez-Cabo F, Lalaguna L, Isern J, Muñoz-Cánoves P, Sabio G, …, Alegre-Cebollada J (2025) Titin cleavage is a driver of cardiomyocyte disengagement and reactive myocardial fibrosis. BioRxiv 2025.04.22.645683. 10.1101/2025.04.22.645683

[CR144] Loreau V, Koolhaas WH, Chan EH, De Boissier P, Brouilly N, Avosani S, Sane A, Pitaval C, Reiter S, Luis NM, Mangeol P, von Philipsborn AC, Rupprecht J-F, Görlich D, Habermann BH, Schnorrer F (2025) Titin-dependent biomechanical feedback tailors sarcomeres to specialized muscle functions in insects. Sci Adv 11(19):eads8716. 10.1126/sciadv.ads8716 40344069 10.1126/sciadv.ads8716 PMC12063666

[CR145] Makarenko I, Opitz CA, Leake MC, Neagoe C, Kulke M, Gwathmey JK, del Monte F, Hajjar RJ, Linke WA (2004) Passive stiffness changes caused by upregulation of compliant titin isoforms in human dilated cardiomyopathy hearts. Circ Res 95(7):708–716. 10.1161/01.RES.0000143901.37063.2f 15345656 10.1161/01.RES.0000143901.37063.2f

[CR146] Marcello M, Cetrangolo V, Savarese M, Udd B (2022) Use of animal models to understand titin physiology and pathology. J Cell Mol Med 26(20):5103–5112. 10.1111/jcmm.17533 36065969 10.1111/jcmm.17533 PMC9575118

[CR147] Martinez-Martin I, Crousilles A, Ochoa JP, Velazquez-Carreras D, Mortensen SA, Herrero-Galan E, Delgado J, Dominguez F, Garcia-Pavia P, de Sancho D, Wilmanns M, Alegre-Cebollada J (2023) Titin domains with reduced core hydrophobicity cause dilated cardiomyopathy. Cell Rep 42(12):113490. 10.1016/j.celrep.2023.113490 38052212 10.1016/j.celrep.2023.113490

[CR148] Mártonfalvi Z, Bianco P, Naftz K, Ferenczy GG, Kellermayer M (2017) Force generation by titin folding. Protein Sci 26(7):1380–1390. 10.1002/pro.3117 28097712 10.1002/pro.3117 PMC5477535

[CR149] Maruyama K (1976) Connectin, an elastic protein from myofibrils. The Journal of Biochemistry 80(2):405–407. 10.1093/oxfordjournals.jbchem.a131291 1002676 10.1093/oxfordjournals.jbchem.a131291

[CR150] Mayans O, Wuerges J, Canela S, Gautel M, Wilmanns M (2001) Structural evidence for a possible role of reversible disulphide bridge formation in the elasticity of the muscle protein titin. Structure. 10.1016/S0969-2126(01)00591-3 11525170 10.1016/s0969-2126(01)00591-3

[CR151] McAfee Q, Caporizzo MA, Uchida K, Bedi KC Jr, Margulies KB, Arany Z, Prosser BL (2023) Truncated titin protein in dilated cardiomyopathy incorporates into the sarcomere and transmits force. J Clin Investig. 10.1172/JCI170196 10.1172/JCI170196 PMC10786684 37943622

[CR152] Methawasin M, Strom JG, Slater RE, Fernandez V, Saripalli C, Granzier H (2016) Experimentally increasing the compliance of titin through RNA binding motif-20 (RBM20) inhibition improves diastolic function in a mouse model of heart failure with preserved ejection fraction. Circulation 134(15):1085–1099. 10.1161/CIRCULATIONAHA.116.023003 27630136 10.1161/CIRCULATIONAHA.116.023003 PMC5069184

[CR153] Methawasin M, Farman GP, Granzier-Nakajima S, Strom J, Kiss B, Smith JE, Granzier H (2022) Shortening the thick filament by partial deletion of titin’s C-zone alters cardiac function by reducing the operating sarcomere length range. J Mol Cell Cardiol 165:103–114. 10.1016/j.yjmcc.2022.01.002 35031281 10.1016/j.yjmcc.2022.01.002 PMC8940690

[CR154] Meurs KM, Friedenberg SG, Kolb J, Saripalli C, Tonino P, Woodruff K, Olby NJ, Keene BW, Adin DB, Yost OL, DeFrancesco TC, Lahmers S, Tou S, Shelton GD, Granzier H (2019) A missense variant in the titin gene in Doberman pinscher dogs with familial dilated cardiomyopathy and sudden cardiac death. Hum Genet 138(5):515–524. 10.1007/s00439-019-01973-2 30715562 10.1007/s00439-019-01973-2

[CR155] Minajeva A, Kulke M, Fernandez JM, Linke WA (2001) Unfolding of titin domains explains the viscoelastic behavior of skeletal myofibrils. Biophys J 80(3):1442–1451. 10.1016/S0006-3495(01)76116-4 11222304 10.1016/S0006-3495(01)76116-4 PMC1301335

[CR156] Morales A, Kinnamon DD, Jordan E, Platt J, Vatta M, Dorschner MO, Starkey CA, Mead JO, Ai T, Burke W, Gastier-Foster J, Jarvik GP, Rehm HL, Nickerson DA, Hershberger RE, Gastier-Foster JM, Bowen DJ, Haas G, Abraham WT, ..., Hindorff L (2020) Variant interpretation for dilated cardiomyopathy.Circ: Genom Precis Med 13(2):43–51. 10.1161/CIRCGEN.119.002480 10.1161/CIRCGEN.119.002480 PMC8070981 32160020

[CR157] Müller AE, Kreiner M, Kötter S, Lassak P, Bloch W, Suhr F, Krüger M (2014) Acute exercise modifies titin phosphorylation and increases cardiac myofilament stiffness. Front Physiol 5(Nov):449. 10.3389/fphys.2014.00449 25477822 10.3389/fphys.2014.00449 PMC4238368

[CR158] Münch J, Abdelilah-Seyfried S (2021) Sensing and responding of cardiomyocytes to changes of tissue stiffness in the diseased heart. Front Cell Dev Biol 9:642840. 10.3389/fcell.2021.642840 33718383 10.3389/fcell.2021.642840 PMC7952448

[CR159] Nagueh SF, Shah G, Wu Y, Torre-Amione G, King NMP, Lahmers S, Witt CC, Becker K, Labeit S, Granzier HL (2004) Altered titin expression, myocardial stiffness, and left ventricular function in patients with dilated cardiomyopathy. Circulation 110(2):155–162. 10.1161/01.CIR.0000135591.37759.AF 15238456 10.1161/01.CIR.0000135591.37759.AF

[CR160] Neagoe C, Kulke M, del Monte F, Gwathmey JK, de Tombe PP, Hajjar RJ, Linke WA (2002) Titin isoform switch in ischemic human heart disease. Circulation 106(11):1333–1341. 10.1161/01.CIR.0000029803.93022.93 12221049 10.1161/01.cir.0000029803.93022.93

[CR161] Neagoe C, Opitz CA, Makarenko I, Linke WA (2003) Gigantic variety: expression patterns of titin isoforms in striated muscles and consequences for myofibrillar passive stiffness. J Muscle Res Cell Motil 24(2–3):175–189. 10.1023/a:1026053530766 14609029 10.1023/a:1026053530766

[CR162] Norton N, Li D, Rampersaud E, Morales A, Martin ER, Zuchner S, Guo S, Gonzalez M, Hedges DJ, Robertson PD, Krumm N, Nickerson DA, Hershberger RE (2013) Exome sequencing and genome-wide linkage analysis in 17 families illustrate the complex contribution of TTN truncating variants to dilated cardiomyopathy. Circ Cardiovasc Genet 6(2):144–153. 10.1161/CIRCGENETICS.111.000062 23418287 10.1161/CIRCGENETICS.111.000062 PMC3815606

[CR163] Oates EC, Jones KJ, Donkervoort S, Charlton A, Brammah S, Smith JE, Ware JS, Yau KS, Swanson LC, Whiffin N, Peduto AJ, Bournazos A, Waddell LB, Farrar MA, Sampaio HA, Teoh HL, Lamont PJ, Mowat D, Fitzsimons RB, Corbett AJ, Ryan MM, O’Grady GL, Sandaradura SA, Ghaoui R, Joshi H, Marshall JL, Nolan MA, Kaur S, Punetha J, Töpf A, Harris E, Bakshi M, Genetti CA, Marttila M, Werlauff U, Streichenberger N, Pestronk A, Mazanti I, Pinner JR, Vuillerot C, Grosmann C, Camacho A, Mohassel P, Leach ME, Foley AR, Bharucha‐Goebel D, Collins J, Connolly AM, Gilbreath HR, Iannaccone ST, Castro D, Cummings BB, Webster RI, Lazaro L, Vissing J, Coppens S, Deconinck N, Luk H-M, Thomas NH, Foulds NC, Illingworth MA, Ellard S, McLean CA, Phadke R, Ravenscroft G, Witting N, Hackman P, Richard I, Cooper ST, Kamsteeg E-J, Hoffman EP, Bushby K, Straub V, Udd B, Ferreiro A, North KN, Clarke NF, Lek M, Beggs AH, Bönnemann CG, MacArthur DG, Granzier H, Davis MR, Laing NG (2018) Congenital titinopathy: comprehensive characterization and pathogenic insights. Ann Neurol 83(6):1105–1124. 10.1002/ana.25241 29691892 10.1002/ana.25241 PMC6105519

[CR164] Opitz CA, Linke WA (2006) Plasticity of cardiac titin/connectin in heart development. J Muscle Res Cell Motil 26(6–8):333–342. 10.1007/s10974-005-9040-7 10.1007/s10974-005-9040-7 16465471

[CR165] Opitz CA, Kulke M, Leake MC, Neagoe C, Hinssen H, Hajjar RJ, Linke WA (2003) Damped elastic recoil of the titin spring in myofibrils of human myocardium. Proc Natl Acad Sci USA 100(22):12688–12693. 10.1073/pnas.2133733100 14563922 10.1073/pnas.2133733100 PMC240679

[CR166] Opitz CA, Leake MC, Makarenko I, Benes V, Linke WA (2004) Developmentally regulated switching of titin size alters myofibrillar stiffness in the perinatal heart. Circ Res 94(7):967–975. 10.1161/01.RES.0000124301.48193.E1 14988228 10.1161/01.RES.0000124301.48193.E1

[CR167] Ottenheijm CAC, Knottnerus AM, Buck D, Luo X, Greer K, Hoying A, Labeit S, Granzier H (2009) Tuning passive mechanics through differential splicing of titin during skeletal muscle development. Biophys J 97(8):2277–2286. 10.1016/j.bpj.2009.07.041 19843460 10.1016/j.bpj.2009.07.041 PMC2764098

[CR168] Ottenheijm CAC, Voermans NC, Hudson BD, Irving T, Stienen GJM, van Engelen BG, Granzier H (2012) Titin-based stiffening of muscle fibers in Ehlers-Danlos syndrome. J Appl Physiol 112(7):1157–1165. 10.1152/japplphysiol.01166.2011 22223454 10.1152/japplphysiol.01166.2011 PMC3774215

[CR169] Palmio J, Leonard-Louis S, Sacconi S, Savarese M, Penttilä S, Semmler AL, Kress W, Mozaffar T, Lai T, Stojkovic T, Berardo A, Reisin R, Attarian S, Urtizberea A, Cobo AM, Maggi L, Kurbatov S, Nikitin S, Milisenda JC, …, Udd B (2019) Expanding the importance of HMERF titinopathy: new mutations and clinical aspects. J Neurol 266(3):680–690. 10.1007/S00415-019-09187-2/FIGURES/2 10.1007/s00415-019-09187-2 PMC6394805 30666435

[CR170] Partanen J, Laulumaa V, Paljärvi L, Partanen K, Naukkarinen A (1994) Late onset foot-drop muscular dystrophy with rimmed vacuoles. J Neurol Sci 125(2):158–167. 10.1016/0022-510X(94)90029-9 7807161 10.1016/0022-510x(94)90029-9

[CR171] Pavel MA, Chen H, Hill M, Sridhar A, Barney M, DeSantiago J, Baskaran A, Owais A, Sandu S, Darbar FA, Ornelas-Loredo A, Al-Azzam B, Chalazan B, Rehman J, Darbar D (2025) A titin missense variant causes atrial fibrillation. In: eLife, vol 14. eLife Sciences Publications Limited. 10.7554/eLife.104719.2

[CR172] Peled Y, Gramlich M, Yoskovitz G, Feinberg MS, Afek A, Polak-Charcon S, Pras E, Sela BA, Konen E, Weissbrod O, Geiger D, Gordon PMK, Thierfelder L, Freimark D, Gerull B, Arad M (2014) Titin mutation in familial restrictive cardiomyopathy. Int J Cardiol 171(1):24–30. 10.1016/J.IJCARD.2013.11.037 24315344 10.1016/j.ijcard.2013.11.037

[CR173] Peng J, Raddatz K, Labeit S, Granzier H, Gotthardt M (2005) Muscle atrophy in titin M-line deficient mice. J Muscle Res Cell Motil 26(6–8):381–388. 10.1007/S10974-005-9020-Y 16470336 10.1007/s10974-005-9020-y

[CR174] Pérez-Vidarte F, Estévez-Arias B, Matalonga L, Yubero D, Codina A, Ortez C, Medina J, DeSena DeCabo L, Carrera-García L, Expósito-Escudero J, Jou C, Tizzano EF, Nascimento A, Natera-de Benito D (2025) Fetal akinesia/hypokinesia and arthrogryposis of neuromuscular origin: etiologic groups, genetics, and phenotypic spectrum. Ann Clin Transl Neurol 12(8):1528–1547. 10.1002/acn3.70088 40443119 10.1002/acn3.70088 PMC12343312

[CR175] Perkin J, Slater R, Del Favero G, Lanzicher T, Hidalgo C, Anderson B, Smith JE, Sbaizero O, Labeit S, Granzier H (2015) Phosphorylating titin’s cardiac N2B element by ERK2 or CaMKIIδ lowers the single molecule and cardiac muscle force. Biophys J 109(12):2592–2601. 10.1016/j.bpj.2015.11.002 26682816 10.1016/j.bpj.2015.11.002 PMC4701010

[CR176] Pernigo S, Fukuzawa A, Bertz M, Holt M, Rief M, Steiner RA, Gautel M (2010) Structural insight into M-band assembly and mechanics from the titin-obscurin-like-1 complex. Proc Natl Acad Sci U S A 107(7):2908–2913. 10.1073/pnas.0913736107 20133654 10.1073/pnas.0913736107 PMC2814874

[CR177] Pfeffer G, Elliott HR, Griffin H, Barresi R, Miller J, Marsh J, Evilä A, Vihola A, Hackman P, Straub V, Dick DJ, Horvath R, Santibanez-Koref M, Udd B, Chinnery PF (2012) Titin mutation segregates with hereditary myopathy with early respiratory failure. Brain 135(6):1695–1713. 10.1093/BRAIN/AWS102 22577215 10.1093/brain/aws102 PMC3359754

[CR178] Pricolo MR, López-Unzu MA, Vicente N, Morales-López C, Huerta-López C, Pérez-Franco W, Dumitru AC, Espinosa FM, Sanchez MI, Garcia R, Silva-Rojas R, Herrero-Galán E, Alegre-Cebollada J (2025) Titin cleavage in living cardiomyocytes induces sarcomere disassembly but does not trigger cell proliferation. BioRxiv. 10.1101/2025.04.22.645658 10.1016/j.jbc.2026.113167 PMC13279373 42142587

[CR179] Puchner EM, Alexandrovich A, Kho AL, Hensen U, Schäfer LV, Brandmeier B, Gräter F, Grubmüller H, Gaub HE, Gautel M (2008) Mechanoenzymatics of titin kinase. Proc Natl Acad Sci U S A 105(36):13385–13390. 10.1073/pnas.0805034105 18765796 10.1073/pnas.0805034105 PMC2527993

[CR180] Radke MH, Peng J, Wu Y, McNabb M, Nelson OL, Granzier H, Gotthardt M (2007) Targeted deletion of titin N2B region leads to diastolic dysfunction and cardiac atrophy. Proc Natl Acad Sci U S A 104(9):3444–3449. 10.1073/pnas.0608543104 17360664 10.1073/pnas.0608543104 PMC1805563

[CR181] Radke MH, Polack C, Methawasin M, Fink C, Granzier HL, Gotthardt M (2019) Deleting full length titin versus the titin M-band region leads to differential mechanosignaling and cardiac phenotypes. Circulation 139(15):1813–1827. 10.1161/CIRCULATIONAHA.118.037588 30700140 10.1161/CIRCULATIONAHA.118.037588 PMC6453709

[CR182] Radke MH, Badillo-Lisakowski V, Britto-Borges T, Kubli DA, Jüttner R, Parakkat P, Carballo JL, Hüttemeister J, Liss M, Hansen A, Dieterich C, Mullick AE, Gotthardt M (2021) Therapeutic inhibition of RBM20 improves diastolic function in a murine heart failure model and human engineered heart tissue. Sci Transl Med. 10.1126/SCITRANSLMED.ABE8952 34851694 10.1126/scitranslmed.abe8952

[CR183] Raskin A, Lange S, Banares K, Lyon RC, Zieseniss A, Lee LK, Yamazaki KG, Granzier HL, Gregorio CC, McCulloch AD, Omens JH, Sheikh F (2012) A novel mechanism involving four-and-a-half LIM domain protein-1 and extracellular signal-regulated kinase-2 regulates titin phosphorylation and mechanics. J Biol Chem 287(35):29273–29284. 10.1074/jbc.M112.372839 22778266 10.1074/jbc.M112.372839 PMC3436149

[CR184] Rees M, Nikoopour R, Fukuzawa A, Kho AL, Fernandez-Garcia MA, Wraige E, Bodi I, Deshpande C, Özdemir Ö, Daimagüler HS, Pfuhl M, Holt M, Brandmeier B, Grover S, Fluss J, Longman C, Farrugia ME, Matthews E, Hanna M, …, Gautel M (2021) Making sense of missense variants in *TTN*-related congenital myopathies. Acta Neuropathol 141(3):431–453. 10.1007/s00401-020-02257-0 10.1007/s00401-020-02257-0 PMC7882473 33449170

[CR185] Rees M, Nikoopour R, Alexandrovich A, Pfuhl M, Lopes LR, Akhtar MM, Syrris P, Elliott P, Carr-White G, Gautel M (2023) Structure determination and analysis of titin A-band fibronectin type III domains provides insights for disease-linked variants and protein oligomerisation. J Struct Biol 215(3):108009. 10.1016/J.JSB.2023.108009 37549721 10.1016/j.jsb.2023.108009 PMC10862085

[CR186] Reichart D, Magnussen C, Zeller T, Blankenberg S (2019) Dilated cardiomyopathy: from epidemiologic to genetic phenotypes: a translational review of current literature. J Intern Med 286(4):362–372. 10.1111/joim.12944 31132311 10.1111/joim.12944

[CR187] Restrepo-Córdoba MA, Chmielewski P, Truszkowska G, Peña-Peña ML, Kubánek M, Krebsová A, Lopes LR, García-Ropero Á, Merlo M, Paldino A, Peters S, Jurcut R, Barriales-Villa R, Zorio E, Hazebroek M, Mogensen J, García-Pavía P (2024) Pregnancy in women with dilated cardiomyopathy genetic variants. Revista Española De Cardiología (English Edition). 10.1016/j.rec.2024.04.002 10.1016/j.rec.2024.04.002 38641168

[CR188] Richards S, Aziz N, Bale S, Bick D, Das S, Gastier-Foster J, Grody WW, Hegde M, Lyon E, Spector E, Voelkerding K, Rehm HL (2015) Standards and guidelines for the interpretation of sequence variants: a joint consensus recommendation of the American College of Medical Genetics and Genomics and the Association for Molecular Pathology. Genet Med. 10.1038/gim.2015.30 25741868 10.1038/gim.2015.30 PMC4544753

[CR189] Rief M, Gautel M, Oesterhelt F, Fernandez JM, Gaub HE (1997) Reversible unfolding of individual titin immunoglobulin domains by AFM. Science 276(5315):1109–1112. 10.1126/science.276.5315.1109 9148804 10.1126/science.276.5315.1109

[CR190] Rivas-Pardo JA, Eckels EC, Popa I, Kosuri P, Linke WA, Fernández JM (2016) Work done by titin protein folding assists muscle contraction. Cell Rep 14(6):1339–1347. 10.1016/j.celrep.2016.01.025 26854230 10.1016/j.celrep.2016.01.025 PMC4865255

[CR191] Rivas-Pardo JA, Li Y, Mártonfalvi Z, Tapia-Rojo R, Unger A, Fernández-Trasancos Á, Herrero-Galán E, Velázquez-Carreras D, Fernández JM, Linke WA, Alegre-Cebollada J (2020) A HaloTag-TEV genetic cassette for mechanical phenotyping of proteins from tissues. Nat Commun 11(1):2060. 10.1038/s41467-020-15465-9 32345978 10.1038/s41467-020-15465-9 PMC7189229

[CR192] Roberts AM, Ware JS, Herman DS, Schafer S, Baksi J, Bick AG, Buchan RJ, Walsh R, John S, Wilkinson S, Mazzarotto F, Felkin LE, Gong S, Macarthur JAL, Cunningham F, Flannick J, Gabriel SB, Altshuler DM, MacDonald PS, …, Cook SA (2015) Integrated allelic, transcriptional, and phenomic dissection of the cardiac effects of titin truncations in health and disease. Sci Transl Med. 10.1126/scitranslmed.3010134 10.1126/scitranslmed.3010134 PMC4560092 25589632

[CR193] Romano R, Ghahremani S, Zimmerman T, Legere N, Thakar K, Ladha FA, Pettinato AM, Hinson JT (2022) Reading frame repair of *TTN* truncation variants restores titin quantity and functions. Circulation 145(3):194–205. 10.1161/CIRCULATIONAHA.120.049997 34905694 10.1161/CIRCULATIONAHA.120.049997 PMC8766920

[CR194] Rudloff MW, Woosley AN, Wright NT (2015) Biophysical characterization of naturally occurring titin M10 mutations. Protein Sci 24(6):946–955. 10.1002/pro.2670 25739468 10.1002/pro.2670 PMC4456108

[CR195] Rudolph F, Hüttemeister J, da Silva Lopes K, Jüttner R, Yu L, Bergmann N, Friedrich D, Preibisch S, Wagner E, Lehnart SE, Gregorio CC, Gotthardt M (2019) Resolving titin’s lifecycle and the spatial organization of protein turnover in mouse cardiomyocytes. Proc Natl Acad Sci U S A 116(50):25126–25136. 10.1073/pnas.1904385116 31757849 10.1073/pnas.1904385116 PMC6911189

[CR196] Rudolph F, Fink C, Hüttemeister J, Kirchner M, Radke MH, Lopez Carballo J, Wagner E, Kohl T, Lehnart SE, Mertins P, Gotthardt M (2020) Deconstructing sarcomeric structure–function relations in titin-BioID knock-in mice. Nat Commun 11(1):3133. 10.1038/s41467-020-16929-8 32561764 10.1038/s41467-020-16929-8 PMC7305127

[CR197] Satoh M, Takahashi M, Sakamoto T, Hiroe M, Marumo F, Kimura A (1999) Structural analysis of the titin gene in hypertrophic cardiomyopathy: identification of a novel disease gene. Biochem Biophys Res Commun 262(2):411–417. 10.1006/bbrc.1999.1221 10462489 10.1006/bbrc.1999.1221

[CR198] Savarese M, Sarparanta J, Vihola A, Udd B, Hackman P (2016) Increasing role of titin mutations in neuromuscular disorders. J Neuromuscul Dis 3(3):293–308. 10.3233/JND-160158 27854229 10.3233/JND-160158 PMC5123623

[CR199] Savarese M, Jonson PH, Huovinen S, Paulin L, Auvinen P, Udd B, Hackman P (2018) The complexity of titin splicing pattern in human adult skeletal muscles. Skelet Muscle 8(1):11. 10.1186/s13395-018-0156-z 29598826 10.1186/s13395-018-0156-z PMC5874998

[CR200] Savarese M, Vihola A, Oates EC, Barresi R, Fiorillo C, Tasca G, Jokela M, Sarkozy A, Luo S, Díaz-Manera J, Ehrstedt C, Rojas-García R, Sáenz A, Muelas N, Lonardo F, Fodstad H, Qureshi T, Johari M, Välipakka S, Udd B (2020) Genotype–phenotype correlations in recessive titinopathies. Genet Med 22(12):2029–2040. 10.1038/s41436-020-0914-2 32778822 10.1038/s41436-020-0914-2

[CR201] Schafer S, de Marvao A, Adami E, Fiedler LR, Ng B, Khin E, Rackham OJL, van Heesch S, Pua CJ, Kui M, Walsh R, Tayal U, Prasad SK, Dawes TJW, Ko NSJ, Sim D, Chan LLH, Chin CWL, Mazzarotto F, Barton PJ, Kreuchwig F, de Kleijn DPV, Totman T, Biffi C, Tee N, Rueckert D, Schneider V, Faber A, Regitz-Zagrosek V, Seidman JG, Seidman CE, Linke WA, Kovalik J-P, O’Regan D, Ware JS, Hubner N, Cook SA (2017) Titin-truncating variants affect heart function in disease cohorts and the general population. Nat Genet 49(1):46–53. 10.1038/ng.3719 27869827 10.1038/ng.3719 PMC5201198

[CR202] Schultheiss HP, Fairweather DL, Caforio ALP, Escher F, Hershberger RE, Lipshultz SE, Liu PP, Matsumori A, Mazzanti A, McMurray J, Priori SG (2019) Dilated cardiomyopathy. Nat Rev Dis Primers 5(1):1–19. 10.1038/s41572-019-0084-1 31073128 10.1038/s41572-019-0084-1 PMC7096917

[CR203] Sheikh F, Raskin A, Chu P-H, Lange S, Domenighetti AA, Zheng M, Liang X, Zhang T, Yajima T, Gu Y, Dalton ND, Mahata SK, Dorn GW, Brown JH, Peterson KL, Omens JH, McCulloch AD, Chen J (2008) An FHL1-containing complex within the cardiomyocyte sarcomere mediates hypertrophic biomechanical stress responses in mice. J Clin Invest 118(12):3870–3880. 10.1172/JCI34472 19033658 10.1172/JCI34472 PMC2575833

[CR204] Shetty NS, Gaonkar M, Pampana A, Patel N, Li P, Arora G, Arora P (2024a) Titin truncating variants, cardiovascular risk factors and the risk of atrial fibrillation and heart failure. Nat Cardiovasc Res. 10.1038/s44161-024-00511-2 39196037 10.1038/s44161-024-00511-2

[CR205] Shetty NS, Pampana A, Patel N, Li P, Arora G, Arora P (2024b) High-proportion spliced-in titin truncating variants in African and European ancestry in the All of Us Research Program. Nat Cardiovasc Res 3(2):140–144. 10.1038/s44161-023-00417-5 39196186 10.1038/s44161-023-00417-5

[CR206] Silva-Rojas R, Vicente N, Gavilán-Herrera M, Labrador-Cantarero V, Sicilia J, Giménez-Sáez O, Dumitru AC, Sánchez MI, Gato-Vilaseca M, Velázquez-Carreras D, López JA, Vázquez J, Herrero-Galán E, López-Unzu MA, Pricolo MR, Alegre-Cebollada J (2025) Mechanically knocking out titin reveals protein tension loss as a trigger of muscle disease. Nat Biomed Eng. 10.1038/s41551-025-01403-x 40473933 10.1038/s41551-025-01403-x

[CR207] Skriver SV, Krett B, Poulsen NS, Krag T, Walas HR, Christensen AH, Bundgaard H, Vissing J, Vissing CR (2023) Skeletal muscle involvement in patients with truncations of titin and familial dilated cardiomyopathy. JACC Heart Fail. 10.1016/j.jchf.2023.10.010 10.1016/j.jchf.2023.10.010 37999665

[CR208] Spracklen TF, Chakafana G, Schwartz PJ, Kotta M-C, Shaboodien G, Ntusi NAB, Sliwa K (2021) Genetics of peripartum cardiomyopathy: current knowledge, future directions and clinical implications. Genes 12(1):103. 10.3390/genes12010103 33467574 10.3390/genes12010103 PMC7830587

[CR209] Squarci C, Bianco P, Reconditi M, Pertici I, Caremani M, Narayanan T, Horváth ÁI, Málnási-Csizmadia A, Linari M, Lombardi V, Piazzesi G (2023) Titin activates myosin filaments in skeletal muscle by switching from an extensible spring to a mechanical rectifier. Proc Natl Acad Sci U S A 120(9):e2219346120. 10.1073/pnas.2219346120 36812205 10.1073/pnas.2219346120 PMC9992839

[CR210] Strom J, Bull M, Gohlke J, Saripalli C, Methawasin M, Gotthardt M, Granzier H (2024) Titin’s cardiac-specific N2B element is critical to mechanotransduction during volume overload of the heart. J Mol Cell Cardiol 191:40–49. 10.1016/J.YJMCC.2024.04.006 38604403 10.1016/j.yjmcc.2024.04.006 PMC11229416

[CR211] Sun Y, Liu X, Huang W, Le S, Yan J (2024) Structural domain in the titin N2B-us region binds to FHL2 in a force-activation dependent manner. Nat Commun 15(1):4496. 10.1038/s41467-024-48828-7 38802383 10.1038/s41467-024-48828-7 PMC11530556

[CR212] Swist S, Unger A, Li Y, Vöge A, von Frieling-Salewsky M, Skärlén Å, Cacciani N, Braun T, Larsson L, Linke WA (2020) Maintenance of sarcomeric integrity in adult muscle cells crucially depends on Z-disc anchored titin. Nat Commun 11(1):4479. 10.1038/s41467-020-18131-2 32900999 10.1038/s41467-020-18131-2 PMC7478974

[CR213] Szent-Györgyi AG (2004) The early history of the biochemistry of muscle contraction. J Gen Physiol 123(6):631–641. 10.1085/JGP.200409091 15173217 10.1085/jgp.200409091 PMC2234565

[CR214] Tamborrini D, Wang Z, Wagner T, Tacke S, Stabrin M, Grange M, Kho AL, Rees M, Bennett P, Gautel M, Raunser S (2023) Structure of the native myosin filament in the relaxed cardiac sarcomere. Nature 623(7988):863–871. 10.1038/s41586-023-06690-5 37914933 10.1038/s41586-023-06690-5 PMC10665186

[CR215] Taylor M, Graw S, Sinagra G, Barnes C, Slavov D, Brun F, Pinamonti B, Salcedo EE, Sauer W, Pyxaras S, Anderson B, Simon B, Bogomolovas J, Labeit S, Granzier H, Mestroni L (2011) Genetic variation in titin in arrhythmogenic right ventricular cardiomyopathy-overlap syndromes. Circulation 124(8):876–885. 10.1161/CIRCULATIONAHA.110.005405 21810661 10.1161/CIRCULATIONAHA.110.005405 PMC3167235

[CR216] Toffali L, Ulivo BD, Giagulli C, Constantin G, Mauri P, Correspondence CL, D’ulivo B, Montresor A, Zenaro E, Delledonne M, Rossato M, Iadarola B, Sbarbati A, Bernardi P, Angelini G, Rossi B, Lopez N, Linke WA, Unger A, …, Laudanna C (2023) An isoform of the giant protein titin is a master regulator of human T lymphocyte trafficking. 10.1016/j.celrep.2023.112516 10.1016/j.celrep.2023.112516 37204926

[CR217] Tomin T, Schittmayer M, Sedej S, Bugger H, Gollmer J, Honeder S, Darnhofer B, Liesinger L, Zuckermann A, Rainer PP, Birner-Gruenberger R (2021) Mass spectrometry-based redox and protein profiling of failing human hearts. Int J Mol Sci 22(4):1787. 10.3390/IJMS22041787 33670142 10.3390/ijms22041787 PMC7916846

[CR218] Tonino P, Kiss B, Strom J, Methawasin M, Smith JE, Kolb J, Labeit S, Granzier H (2017) The giant protein titin regulates the length of the striated muscle thick filament. Nat Commun 8(1):1–11. 10.1038/s41467-017-01144-9 29051486 10.1038/s41467-017-01144-9 PMC5648799

[CR219] Tonino P, Kiss B, Gohlke J, Smith JE, Granzier H (2019) Fine mapping titin’s C-zone: matching cardiac myosin-binding protein C stripes with titin’s super-repeats. J Mol Cell Cardiol 133:47–56. 10.1016/j.yjmcc.2019.05.026 31158359 10.1016/j.yjmcc.2019.05.026 PMC6639027

[CR220] Töpf A, Cox D, Zaharieva IT, Di Leo V, Sarparanta J, Jonson PH, Sealy IM, Smolnikov A, White RJ, Vihola A, Savarese M, Merteroglu M, Wali N, Laricchia KM, Venturini C, Vroling B, Stenton SL, Cummings BB, Harris E, ..., Straub V (2024) Digenic inheritance involving a muscle-specific protein kinase and the giant titin protein causes a skeletal muscle myopathy. Nat Gen 56(3):395–407. 10.1038/s41588-023-01651-0 10.1038/s41588-023-01651-0 PMC10937387 38429495

[CR221] Tskhovrebova L, Trinick J, Sleep JA, Simmons RM (1997) Elasticity and unfolding of single molecules of the giant muscle protein titin. Nature 387(6630):308–312. 10.1038/387308a0 9153398 10.1038/387308a0

[CR222] Tskhovrebova L, Bennett P, Gautel M, Trinick J (2015) Titin ruler hypothesis not refuted. Proc Natl Acad Sci U S A 112(11):E1172. 10.1073/pnas.1422326112 25695970 10.1073/pnas.1422326112 PMC4371926

[CR223] Uchida K, Scarborough EA, Prosser BL (2022) Cardiomyocyte microtubules: control of mechanics, transport, and remodeling. Annu Rev Physiol 84(1):257–283. 10.1146/annurev-physiol-062421-040656 34614374 10.1146/annurev-physiol-062421-040656 PMC9097619

[CR224] Udd B, Kääriänen H, Somer H (1991) Muscular dystrophy with separate clinical phenotypes in a large family. Muscle Nerve 14(11):1050–1058. 10.1002/MUS.880141103 1745277 10.1002/mus.880141103

[CR225] Udd B, Vihola A, Sarparanta J, Richard I, Hackman P (2005) Titinopathies and extension of the M-line mutation phenotype beyond distal myopathy and LGMD2J. Neurology 64(4):636–642. 10.1212/01.WNL.0000151853.50144.82 15728284 10.1212/01.WNL.0000151853.50144.82

[CR226] Vahle B, Schauer A, Augstein A, Jarabo M-EP, Friedrich J, Barthel P, Männel A, Mangner N, Labeit S, Bowen TS, Linke A, Adams V (2025) MyoMed205 counteracts titin hyperphosphorylation and the expression of contraction-regulating proteins in a rat model of HFpEF. J Cachexia Sarcopenia Muscle 16(3):e13843. 10.1002/jcsm.13843 40464169 10.1002/jcsm.13843 PMC12134774

[CR227] van der Pijl R, Strom J, Conijn S, Lindqvist J, Labeit S, Granzier H, Ottenheijm C (2018) Titin-based mechanosensing modulates muscle hypertrophy. J Cachexia Sarcopenia Muscle 9(5):947–961. 10.1002/jcsm.12319 29978560 10.1002/jcsm.12319 PMC6204599

[CR228] van der Pijl RJ, van den Berg M, van de Locht M, Shen S, Bogaards SJP, Conijn S, Langlais P, Hooijman PE, Labeit S, Heunks LMA, Granzier H, Ottenheijm CAC (2021) Muscle ankyrin repeat protein 1 (MARP1) locks titin to the sarcomeric thin filament and is a passive force regulator. J Gen Physiol. 10.1085/jgp.202112925 34152365 10.1085/jgp.202112925 PMC8222902

[CR229] van der Pijl R, Gohlke J, Strom J, Peters E, Shen S, Conijn S, Hourani Z, Lange S, Chen J, Langlais P, Labeit S, Granzier H, Ottenheijm C (2025) The titin N2A-MARP signalosome constrains muscle longitudinal hypertrophy in response to stretch. BioRxiv Preprint Serv Biol 14. 10.1101/2025.06.19.660595 10.7554/eLife.107597 PMC13423355 42530086

[CR230] van Heerebeek L, Hamdani N, Falcão-Pires I, Leite-Moreira AF, Begieneman MPV, Bronzwaer JGF, van der Velden J, Stienen GJM, Laarman GJ, Somsen A, Verheugt FWA, Niessen HWM, Paulus WJ (2012) Low myocardial protein kinase G activity in heart failure with preserved ejection fraction. Circulation 126(7):830–839. 10.1161/CIRCULATIONAHA.111.076075 22806632 10.1161/CIRCULATIONAHA.111.076075

[CR231] van Heesch S, Witte F, Schneider-Lunitz V, Schulz JF, Adami E, Faber AB, Kirchner M, Maatz H, Blachut S, Sandmann C-L, Kanda M, Worth CL, Schafer S, Calviello L, Merriott R, Patone G, Hummel O, Wyler E, Obermayer B, Mücke MB, Lindberg EL, Trnka F, Memczak S, Schilling M, Felkin LE, Barton PJR, Quaife NM, Vanezis K, Diecke S, Mukai M, Mah N, Oh S-J, Kurtz A, Schramm C, Schwinge D, Sebode M, Harakalova M, Asselbergs FW, Vink A, de Weger RA, Viswanathan S, Widjaja AA, Gärtner-Rommel A, Milting H, dos Remedios C, Knosalla C, Mertins P, Landthaler M, Vingron M, Linke WA, Seidman JG, Seidman CE, Rajewsky N, Ohler U, Cook SA, Hubner N (2019) The translational landscape of the human heart. Cell 178(1):242-260.e29. 10.1016/j.cell.2019.05.010 31155234 10.1016/j.cell.2019.05.010

[CR232] Van Spaendonck-Zwarts KY, Posafalvi A, Van Den Berg MP, Hilfiker-Kleiner D, Bollen IAE, Sliwa K, Alders M, Almomani R, Van Langen IM, Van Der Meer P, Sinke RJ, Van Der Velden J, Van Veldhuisen DJ, Van Tintelen JP, Jongbloed JDH (2014) Titin gene mutations are common in families with both peripartum cardiomyopathy and dilated cardiomyopathy. Eur Heart J 35(32):2165–2173. 10.1093/EURHEARTJ/EHU050 24558114 10.1093/eurheartj/ehu050

[CR233] Vatta M, Regalado E, Parfenov M, Swartzlander D, Nagl A, Mannello M, Lewis R, Clemens D, Garcia J, Ellsworth RE, Morales A, Ting YL, Aradhya S (2025) Analysis of TTN truncating variants in >74 000 cases reveals new clinically relevant gene regions. Circ Genom Precis Med 18(2). 10.1161/CIRCGEN.124.004982 10.1161/CIRCGEN.124.004982 PMC11999099 39968638

[CR234] Verdonschot JAJ, Hazebroek MR, Derks KWJ, Barandiarán Aizpurua A, Merken JJ, Wang P, Bierau J, Van Den Wijngaard A, Schalla SM, Abdul Hamid MA, Van Bilsen M, Van Empel VPM, Knackstedt C, Brunner-La Rocca HP, Brunner HG, Krapels IPC, Heymans SRB (2018) Titin cardiomyopathy leads to altered mitochondrial energetics, increased fibrosis and long-term life-threatening arrhythmias. Eur Heart J 39(10):864–873. 10.1093/EURHEARTJ/EHX808 29377983 10.1093/eurheartj/ehx808

[CR235] Von Castelmur E, Marino M, Svergun DI, Kreplak L, Ucurum-Fotiadis Z, Konarev PV, Urzhumtsev A, Labeit D, Labeit S, Mayans O (2008) A regular pattern of Ig super-motifs defines segmental flexibility as the elastic mechanism of the titin chain. Proc Natl Acad Sci U S A 105(4):1186–1191. 10.1073/PNAS.0707163105 18212128 10.1073/pnas.0707163105 PMC2234113

[CR236] Walsh R, Thomson KL, Ware JS, Funke BH, Woodley J, McGuire KJ, Mazzarotto F, Blair E, Seller A, Taylor JC, Minikel EV, Exome Aggregation Consortium, MacArthur DG, Farrall M, Cook SA, Watkins H (2017) Reassessment of Mendelian gene pathogenicity using 7,855 cardiomyopathy cases and 60,706 reference samples. Genet Med 19(2):192–203. 10.1038/gim.2016.90 10.1038/gim.2016.90 PMC5116235 27532257

[CR237] Wang K, McClure J, Tu A (1979) Titin: major myofibrillar components of striated muscle. Proc Natl Acad Sci 76(8):3698–3702. 10.1073/pnas.76.8.3698 291034 10.1073/pnas.76.8.3698 PMC383900

[CR238] Ware JS, Cook SA (2018) Role of titin in cardiomyopathy: from DNA variants to patient stratification. Nat Rev Cardiol. 10.1038/nrcardio.2017.190 29238064 10.1038/nrcardio.2017.190

[CR239] Ware JS, Li J, Mazaika E, Yasso CM, DeSouza T, Cappola TP, Tsai EJ, Hilfiker-Kleiner D, Kamiya CA, Mazzarotto F, Cook SA, Halder I, Prasad SK, Pisarcik J, Hanley-Yanez K, Alharethi R, Damp J, Hsich E, Elkayam U (2016) Shared genetic predisposition in peripartum and dilated cardiomyopathies. N Engl J Med 374(3):233–241. 10.1056/NEJMoa1505517 26735901 10.1056/NEJMoa1505517 PMC4797319

[CR240] Ware JS, Amor-Salamanca A, Tayal U, Govind R, Serrano I, Salazar-Mendiguchía J, García-Pinilla JM, Pascual-Figal DA, Nuñez J, Guzzo-Merello G, Gonzalez-Vioque E, Bardaji A, Manito N, López-Garrido MA, Padron-Barthe L, Edwards E, Whiffin N, Walsh R, Buchan RJ, Midwinter W, Wilk A, Prasad S, Pantazis A, Baski J, O’Regan DP, Alonso-Pulpon L, Cook SA, Lara-Pezzi E, Barton PJ, Garcia-Pavia P (2018) Genetic etiology for alcohol-induced cardiac toxicity. J Am Coll Cardiol 71(20):2293–2302. 10.1016/j.jacc.2018.03.462 29773157 10.1016/j.jacc.2018.03.462 PMC5957753

[CR241] Warren CM, Krzesinski PR, Campbell KS, Moss RL, Greaser ML (2004) Titin isoform changes in rat myocardium during development. Mech Dev 121(11):1301–1312. 10.1016/j.mod.2004.07.003 15454261 10.1016/j.mod.2004.07.003

[CR242] Watanabe D, Mishima T, Hamada T (2025) Reversible oxidative modifications partially cause myofibrillar active and passive force decline in early phase of immobilization. Am J Physiol Cell Physiol 329(3):C939–C952. 10.1152/AJPCELL.00554.2025 40857174 10.1152/ajpcell.00554.2025

[CR243] Weinert S, Bergmann N, Luo X, Erdmann B, Gotthardt M (2006) M line–deficient titin causes cardiac lethality through impaired maturation of the sarcomere. J Cell Biol 173(4):559–570. 10.1083/JCB.200601014 16702235 10.1083/jcb.200601014 PMC2063865

[CR244] Weintraub RG, Semsarian C, Macdonald P (2017) Dilated cardiomyopathy. Lancet 390(10092):400–414. 10.1016/S0140-6736(16)31713-5 28190577 10.1016/S0140-6736(16)31713-5

[CR245] Weston T, Rees M, Gautel M, Fraternali F (2024) Walking with giants: the challenges of variant impact assessment in the giant sarcomeric protein titin. Wires Mech Dis 16(2):e1638. 10.1002/wsbm.1638 38155593 10.1002/wsbm.1638

[CR246] Weston T, Ng J, Gracia Carmona O, Gautel M, Fraternali F (2025) TITINdb2-expanding annotation and structural information for protein variants in the giant sarcomeric protein titin. Bioinform Adv 5(1):vbaf062. 10.1093/bioadv/vbaf062 40270927 10.1093/bioadv/vbaf062 PMC12017618

[CR247] Witt CC, Ono Y, Puschmann E, McNabb M, Wu Y, Gotthardt M, Witt SH, Haak M, Labeit D, Gregorio CC, Sorimachi H, Granzier H, Labeit S (2004) Induction and myofibrillar targeting of CARP, and suppression of the Nkx2.5 pathway in the MDM mouse with impaired titin-based signaling. J Mol Biol 336(1):145–154. 10.1016/j.jmb.2003.12.021 14741210 10.1016/j.jmb.2003.12.021

[CR248] Yamasaki R, Wu Y, McNabb M, Greaser M, Labeit S, Granzier H (2002) Protein kinase A phosphorylates titin’s cardiac-specific N2B domain and reduces passive tension in rat cardiac myocytes. Circ Res 90(11):1181–1188. 10.1161/01.RES.0000021115.24712.99 12065321 10.1161/01.res.0000021115.24712.99

[CR249] Yu M, Lu J-H, Le S, Yan J (2021) Unexpected low mechanical stability of titin I27 domain at physiologically relevant temperature. J Phys Chem Lett 12(33):7914–7920. 10.1021/acs.jpclett.1c01309 34384021 10.1021/acs.jpclett.1c01309

[CR250] Zastrow MS, Flaherty DB, Benian GM, Wilson KL (2006) Nuclear titin interacts with A- and B-type lamins in vitro and in vivo. J Cell Sci 119(2):239–249. 10.1242/JCS.02728 16410549 10.1242/jcs.02728

[CR251] Zaunbrecher RJ, Abel AN, Beussman K, Leonard A, Von Frieling-Salewsky M, Fields PA, Pabon L, Reinecke H, Yang X, MacAdangdang J, Kim DH, Linke WA, Sniadecki NJ, Regnier M, Murry CE (2019) Cronos titin is expressed in human cardiomyocytes and necessary for normal sarcomere function. Circulation 140(20):1647–1660. 10.1161/CIRCULATIONAHA.119.039521 31587567 10.1161/CIRCULATIONAHA.119.039521 PMC6911360

[CR252] Zhou T, Fleming JR, Lange S, Hessel AL, Bogomolovas J, Stronczek C, Grundei D, Ghassemian M, Biju A, Börgeson E, Bullard B, Linke WA, Chen J, Kovermann M, Mayans O (2021) Molecular characterisation of titin N2A and its binding of CARP reveals a titin/actin cross-linking mechanism. J Mol Biol 433(9):166901. 10.1016/j.jmb.2021.166901 33647290 10.1016/j.jmb.2021.166901 PMC8052292

[CR253] Zhu Y, Bogomolovas J, Labeit S, Granzier H (2009) Single molecule force spectroscopy of the cardiac titin N2B element. J Biol Chem 284(20):13914–13923. 10.1074/jbc.M809743200 19282282 10.1074/jbc.M809743200 PMC2679491

[CR254] Zhu C, Yin Z, Ren J, McCormick RJ, Ford SP, Guo W (2015) RBM20 is an essential factor for thyroid hormone-regulated titin isoform transition. J Mol Cell Biol 7(1):88. 10.1093/JMCB/MJV002 25573899 10.1093/jmcb/mjv002 PMC4400401

[CR255] Zhu C, Yin Z, Tan B, Guo W (2017) Insulin regulates titin pre-mRNA splicing through the PI3K-Akt-mTOR kinase axis in a RBM20-dependent manner. Biochimica Et Biophysica Acta (BBA) - Molecular Basis of Disease 1863(9):2363–2371. 10.1016/j.bbadis.2017.06.023 28676430 10.1016/j.bbadis.2017.06.023 PMC5547897

[CR256] Zou J, Tran D, Baalbaki M, Tang LF, Poon A, Pelonero A, Titus EW, Yuan C, Shi C, Patchava S, Halper E, Garg J, Movsesyan I, Yin C, Wu R, Wilsbacher LD, Liu J, Hager RL, Coughlin SR, Jinek M, Pullinger CR, Kane JP, Hart DO, Kwok P-Y, Deo RC (2015) An internal promoter underlies the difference in disease severity between N- and C-terminal truncation mutations of titin in zebrafish. Elife 4:e09406. 10.7554/eLife.09406 26473617 10.7554/eLife.09406 PMC4720518

[CR257] Zuo J, Zhan D, Xia J, Li H (2021) Single-molecule force spectroscopy studies of missense titin mutations that are likely causing cardiomyopathy. Langmuir 37(41):12128–12137. 10.1021/acs.langmuir.1c02006 34618459 10.1021/acs.langmuir.1c02006 PMC9150697

